# Single low dose primaquine to block the transmission of *Plasmodium falciparum*—proposed stand-alone and ACT-adapted regimens

**DOI:** 10.1186/s12916-025-04153-4

**Published:** 2025-07-01

**Authors:** Walter R. J. Taylor, Peter Olupot-Olupot, Marie A. Onyamboko, Natenapa Chimjinda, Chiraporn Taya, Julie Nguyen Ngoc Pouplin, Thomas N. Williams, Kathryn Maitland, Caterina A. Fanello, Nicholas P. J. Day, Joel Tarning, Nicholas J. White, Mavuto Mukaka

**Affiliations:** 1 Mahidol Oxford Tropical Medicine Research Unit (MORU), Faculty of Tropical Medicine, Mahidol University, 420/6 Rajvithi Road, Bangkok, 10400 Thailand; 2 Centre for Tropical Medicine and Global Health, Nuffield Department of Medicine, University of Oxford, Oxford, UK; 3 Mbale Clinical Research Institute (MCRI), P.O. Box 1966, Mbale, Uganda; 4 Busitema University, P.O. Box 1460, Mbale, Uganda; 5 Kinshasa School of Public Health, University of Kinshasa, Avenue Tombalbaye 68-78, Kinshasa, Democratic Republic of Congo; 6 Réseau Médicaments & Développement (ReMeD), 21B Avenue du Commandant L’Herminier, St. Nazaire, France; 7 KEMRI-Wellcome Trust Research Programme, Kilifi, Kenya; 8 Institute of Global Health Innovation, Department of Surgery and Cancer, Imperial College London, London, SW7 2 AS UK

**Keywords:** Primaquine, Transmission blocking, *Plasmodium falciparum*, Allometric dosing, Haemolysis, Glucose-6-phosphate dehydrogenase deficiency

## Abstract

**Background:**

Despite the 2012 WHO recommendation to add single low dose primaquine (SLDPQ, 0.25 mg/kg body weight) to artemisinin-based combination treatments (ACTs) for blocking the transmission of artemisinin-resistant *Plasmodium falciparum*, there are currently no weight-based regimens founded on robust evidence.

**Methods:**

Applying published safety, transmission blocking and pharmacokinetic data, and exploring pharmacokinetic-pharmacodynamic relationships of age-based dosing of SLDPQ in African children with acute, uncomplicated *Plasmodium falciparum*, we derived weight-based, stand-alone, ACT-, triple ACT-, and vivax-matched regimens by following allometric dosing principles and simulating PQ exposure (area under the concentration time curve). The ACTs were dihydroartemisinin piperaquine (DHAPP), artesunate pyronaridine (ASPYR), artesunate amodiaquine (ASAQ), artesunate mefloquine (ASMQ), artemether lumefantrine (AL), and ALAQ. Tablet strengths were predefined: 2.5, 3.75, 5, 7.5, and 15 mg, and no tablet fractions were allowed. The maximum mg/kg dose was set at 0.5, and, primarily for ease of ACT co-blistering, 1 tablet = 1 dose. We assessed different mg/kg doses and selected the dosing associated with a predicted median exposure closest to 1200 ng*h/mL, the exposure predicted for a 60 kg individual given 15 mg of PQ.

**Results:**

The designed 8 regimens had 4–8 dosing bands. The stand-alone, DHAPP, and ASPYR regimens contain the full line of PQ tablets and all other regimens, except AL (2.5, 7.5, 15 mg) and ALAQ (2.5, 5, 7.5, 15 mg), use 3.75 mg. The 2.5 mg tablet resulted in a maximum dose of 0.56 mg/kg for ASAQ, as this regimen starts at 4.5 kg body weight, whilst all other regimens start at 5 kg and resulted in 0.5 mg/kg. Substituting 3.75 mg with 5 mg results in maximum doses of 0.56 mg/kg (ASAQ, ASMQ) and 0.63 mg/kg (other regimens), risking greater toxicity. Across all dosing bands, 0.17 − 0.56 mg/kg doses predict exposures of ~ 500 − 2000 ng*mL/h. Regimens with more dosing bands had less variations in exposure.

**Conclusions:**

These regimens offer flexibility for malaria control programmes and guidance for drug manufacturers wishing to co-blister SLDPQ with ACTs. The WHO should reinstate the 3.75 mg tablet for prequalification and determine which regimens should be incorporated into their treatment guidelines to advance malaria elimination.

**Trial registration:**

The trial is registered at ISRCTN, number 11594437.

**Supplementary Information:**

The online version contains supplementary material available at 10.1186/s12916-025-04153-4.

## Background

In order to counter the threat of artemisinin-resistant *Plasmodium falciparum* that was emerging in the Mekong subregion, the WHO recommended in 2012 the addition of single low dose primaquine (SLDPQ, 0.25 mg/kg body weight) to block the transmission of resistant parasites and, if deployed widely, SLDPQ would benefit malaria endemic communities [1]. The optimal dose remains elusive despite a body of research that includes dose ranging studies and weight- and aged-based regimens [2–7].

An aged-based regimen for Africa was proposed in 2018 [8], intending to achieve transmission blocking efficacy with safety in glucose-6-phopshate dehydrogenase deficient (G6PDd) individuals. Based on limited evidence at the time, suggested therapeutic dose ranges were 0.15–0.5 mg PQ base/kg body weight for individuals aged ≥ 6 years and 0.15–0.4 mg PQ base/kg for children aged 1–5 years, given their greater propensity to haemolyse compared to older children and adults when treated for malaria [9]. For children aged 6 months– < 1 year, a cautious 1.25 mg dose was suggested.

Using 2.5, 5, 7.5, and 15 mg tablets, this age-based regimen was tested in > 560 children aged < 12 years in a placebo-controlled trial of 1137 patients that included 284 G6PD deficient boys and girls and 119 heterozygous girls [6]. Reassuringly, SLDPQ had the same tolerability and transfusion rate as placebo (~ 1%), consistent with transfusion rates in other settings of ~ 1–1.5% [10–12]. Moreover, receiving SLDPQ and being G6PD deficient were not independently associated with any deleterious post-treatment effects on the initial absolute and factional fall in haemoglobin (Hb), the nadir Hb concentration, and Hb recovery. Indeed, compared to G6PD normal children, G6PDd children had a more robust reticulocyte response and a higher mean day 42 Hb concentration, especially, in those with low baseline Hb concentrations [13].

PQ disposition was characterised by wide interindividual variation in maximum concentrations (*C*_max_) and exposures [area under the drug concentration time curve (AUC)] as well as an increased body weight-normalised elimination clearance (Additional file 1: Fig. S1) that peaked at ~ 18 months (median wt 10 kg) and plateaued at 6 years (median wt 17.5 kg) for a median fold difference of 1.6 [14].

For malaria control programmes (MCPs), SLDPQ safety remains troubling despite growing evidence of the very low risk of clinically significant haemolysis [5, 15–20]. In their systematic review of mostly falciparum-infected African patients [15], Stepniewska et al. reported that an increase of 0.1 mg/kg in PQ in 194 G6PDd African individuals (presumed G6PD A− , median age 21 years, IQR 12–30) resulted in a mean Hb decrease of 0.27 (95% CI 0.19–0.34) g/dL; the predicted median Hb falls were -2.9 vs. -1.3 g/dL in 0.75 and 0.25 mg/kg recipients, respectively. This meta-analysis supports the WHO-recommended target dose of 0.25 mg/kg but was hampered by only half of the studies administering PQ on D0 (~ 16%) or D1 (~ 34%), and the number of children < 5 years with G6PDd was small: 28 from Africa and 21 from Asia.

Increasing experience with the 0.75 mg/kg target dose in severe SE Asian G6PD variants in vivax patients remains mostly reassuring [21], despite one transfusion in a G6PDd Cambodian male [22], as well as data from Shekalaghe et al. in asymptomatic Tanzanian children aged ≤ 12 years [23]. A significant minority received > 1 mg/kg [8] but the resulting haemolysis (mean fall 2.5 g/dL) in the G6PDd children was well tolerated.

Gametocyte carriage is a surrogate marker of infectivity and gametocytaemia has a sigmoidal relationship to infectivity; thus, transmission blocking efficacy is best assessed by mosquito infectivity studies [24]. PQ and its predecessor, plasmoquine, rapidly reduce mosquito infectivity, often within 24 h [2, 25–28], with two studies suggesting similar effectiveness of 15, 30, and 45 mg [27, 28]. However, Chotsiri et al. quantified the dose–response relationship [29] in predominantly asymptomatic, older (median age 12 years), G6PD normal, falciparum-infected males [2]. The predicted median times to no infected mosquitoes were ~ 17 (placebo), ~ 2.5 (0.0625 mg/kg), and 1.5 (0.125 mg/kg) days vs. ~ 17 and ~ 10 h for 0.25 and 0.5 mg/kg, respectively. These data show a clinically small difference in efficacy between the two highest doses and support the WHO-recommended, PQ target dose of 0.25 mg/kg.

Hitherto, safety and PK data have been lacking in young, G6PDd African children with acute uncomplicated falciparum malaria, a key target group. Having closed this gap, we embarked on designing SDLPQ regimens, with an emphasis on tolerability, at a time of the rapid spread of artemisinin resistance in eastern Africa [30–34].

## Methods

### Study details

Full descriptions of study details are explained elsewhere [6, 14]. Briefly, we gave age-based doses of SLDPQ/placebo to Ugandan and Congolese children, aged 6 months–11 years with acute uncomplicated falciparum malaria and a Hb ≥ 6 g/dL. Genotyping characterised G6PD, alpha thalassaemia, sickle cell, and cytochrome (CYP) P450 2D6 metaboliser status (PK substudy, *n* = 250). The study took place between July 2017 and November 2019 in Mbale, eastern Uganda, and Kinshasa, Democratic Republic of Congo (DRC), and was approved by all pertinent ethics committees. In the 250 patient subset, Mukaka et al. performed a non-compartmental analysis to estimate the pharmacokinetic parameters and then assessed the independent factors affecting those parameters, in particular, primaquine exposure (AUC_0-last_) [14]. The trial was registered on the 9th of May 2017 at the ISRCTN, number 11594437.

### Pharmacokinetic-pharmacodynamic relationship

Through scatter plots and simple and multivariable linear regressions, we examined the relationships between PQ mg/kg dose, *C*_max_, and AUC_0-last_ [14] and (i) Hb fractional fall (%): 100 × (nadir Hb − baseline Hb)/baseline Hb, (ii) peak methaemoglobin (metHb) from D1–3, (iii) gametocyte carriage over time, and (iv) the percent reduction in gametocytaemia (gam ct, Eq. 1) as a pharmacodynamic (PD) surrogate marker of transmission blocking efficacy.

1 $$100\times\frac{\left(\text{gam ct D}2-\text{gam ct D}0\right)}{\text{gam ct D}0}\%$$

### Pharmacokinetic model to predict PQ exposure

**Fig. 1:**
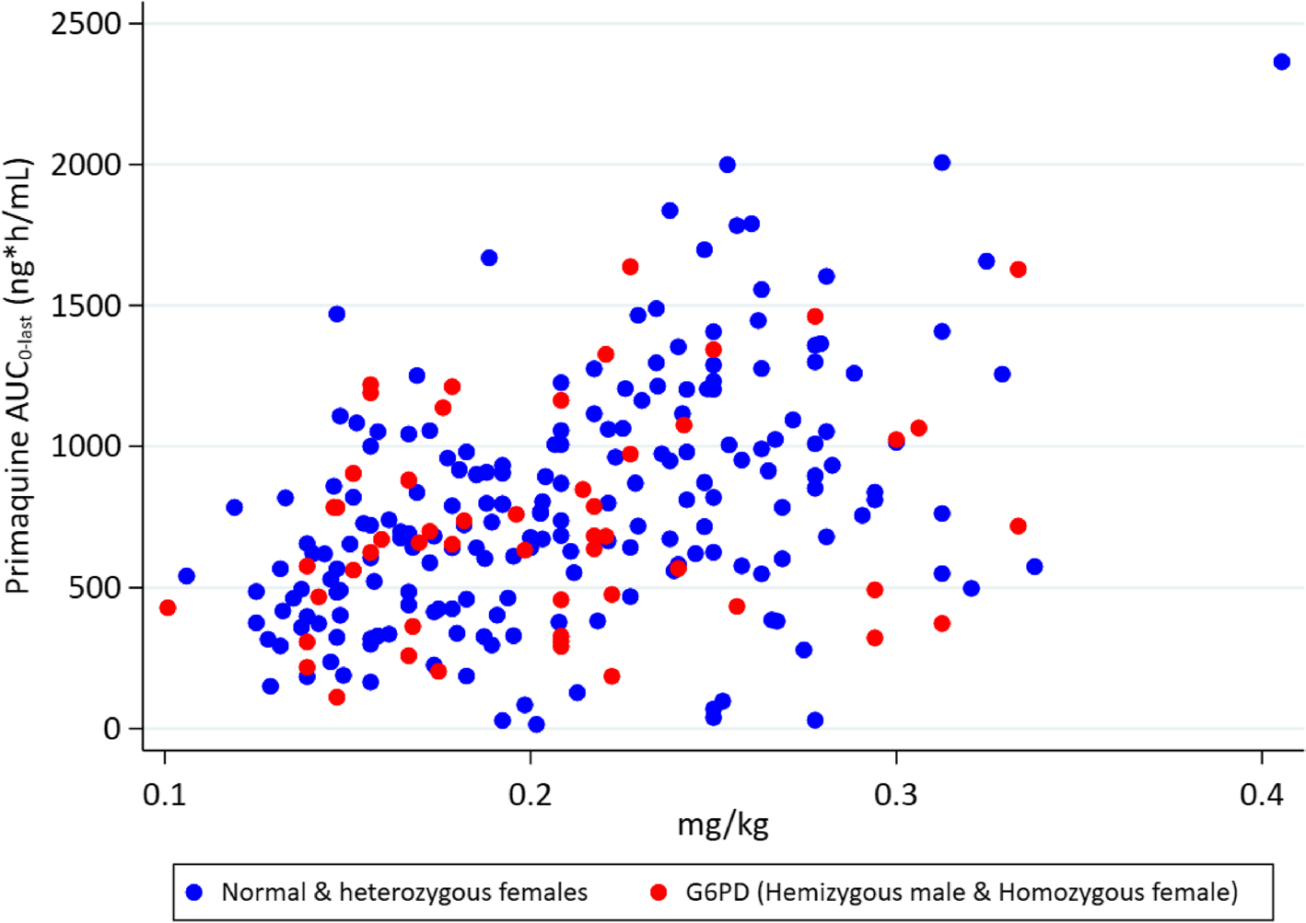
Measured primaquine exposure as a function of the mg/kg dose administered in G6PD deficient children and a combination of G6PD heterozygous females and normal children

Following age-dosed PQ, resulting in mg/kg doses of 0.07 to 0.41, 45% (113/250) of the measured AUCs fell between 500 and 1000 ng*h/mL, 27% (*n* = 70) were > 1000 ng*h/mL, leaving 28% < 500 ng*h/mL (Fig. 1) [14].

Although these exposures resulted in good safety and a significant reduction in gametocyte carriage vs. placebo [6], we hypothesise that optimal exposures would be achieved by tighter dosing around the 0.25 mg/kg target dose by using weight-based regimens.

Four covariates were independently associated with PQ exposure: mg/kg dose, age, baseline Hb, and the CYP 2D6 activity score (AS) [14], resulting in Eq. 2 below.

2 $$\text{AUC}=590+(3050\times \text{mg}/\text{kg dose of PQ})+(22\times \text{age in years})-(30\times \text{D}0\text{Hb g}/\text{dL})-(166\times \text{AS})$$

This equation predicts that, on average, for every 0.1 mg/kg dose increase, the AUC will increase by 305 ng*h/mL, and by 22 ng*h/mL for every 1-year increase in age whilst increases of 1 g/dL of Hb at presentation and one unit of AS will decrease the AUC by 30 and 166 ng*h/mL, respectively.

This AUC model is validated only for children aged 6 months– < 12 years and the goodness of fit analysis showed a good correlation between observed and predicted AUC_0-last_ values, with no trends in residual plots (Additional file 1: Fig. S2). However, we have gone beyond the model limits and report the AUCs across the weight spectrum from 4.5 to 90 kg and capped the age at 18 years. Accordingly, an 18 years old, 60 kg person dosed with 15 mg of primaquine and with a Hb of 10 g/dL and an AS of 1.5 has a predicted AUC of 1200 ng*h/mL. Given the dose proportional pharmacokinetics of primaquine [35, 36], this predicted AUC is consistent with the calculated median AUC_0–24_ of 4311 ng*h/mL when 45 mg was given to 7 *P. falciparum* patients (mean weight 51 kg, mean PQ dose = 0.88 mg/kg) [37], and to the median AUC_0–24_ of 1090 − 1130 ng*h/mL reported in healthy Thai adults given 30 mg [38–40]. The observed exposure differences between studies suggest a disease effect on PQ disposition and the data from Edwards et al. support our prediction formula as being roughly applicable to adults with falciparum malaria [37].

### Dose selection

We prospectively defined several restrictions/parameters. The tablet strengths for testing were 2.5, 3.75, 5, 7.5, and 15 mg. No tablet fractions were allowed, and 1 tablet would equal 1 dose (primarily for ACT co-blistering) but using two PQ tablet strengths was also explored. The stand-alone regimen (i.e. independent of an ACT/triple ACT) used the full line of PQ tablet strengths and includes a 22.5 mg dose for the heavier individuals, resulting in 6 dosing bands. We also explored replacing 3.75 mg with 5 mg in case the 3.75 mg tablet is not available in the future. PQ safety was favoured over PQ transmission blocking efficacy and so 0.5 mg/kg of PQ was set as the maximum dose. However, this was not possible for the ASAQ regimen because it starts at 4.5 kg, resulting in 0.56 mg/kg with the lowest tablet strength.

Dosing was also designed to match commonly used ACTs (4 − 8 dosing bands): (i) artemether lumefantrine (AL, 4 bands), artesunate amodiaquine (ASAQ, 4 bands), artesunate mefloquine (ASMQ, 4 bands), artesunate pyronaridine (ASPYR, 7 bands), dihydroartemisinin piperaquine (DHAPP, 8 bands), (ii) a new triple ACT, ALAQ (5 bands), and (iii) a proposed antirelapse regimen for *P. vivax* infections (5 bands) [41].

A 700,000+ African anthropometric database [8] was used to define weight-age relationships, selecting the median ages for each 1 kg interval from 5 to 90 kg. In all dose simulations, we assumed a baseline Hb of 10 g/dL, an AS of 1.5, i.e. a normal CYP 2D6 metaboliser (AS = 1.25–2.25 [42]), which was the majority metaboliser status in the PK substudy, 150/250 (60%), and that all weights above 38 kg were associated with an age variable of 18 years. Different doses were evaluated for each body weight by predicting individual exposures from Eq. 2. The dose generating a median exposure in the dosing band closest to 1200 ng*h/mL was deemed the most appropriate dose for that particular weight band.

A higher mg/kg dose is needed in younger children to compensate for their relatively higher clearance/kg and achieve comparable exposures to adults. Therefore, we also estimated the mg/kg dose in children according to the allometric scale equation below (Eq. 3, BW = body weight) [43] and used a target adult dose of 15 mg of PQ in a 60 kg person (0.25 mg/kg). The allometric line was plotted against the proposed mg/kg doses achieved by the selected dosing regimens.

3 $$\text{PQ dose }\left(\text{mg}\right)=15\text{ mg}\times {\left(\frac{\text{BW}}{60}\right)}^{0.75}$$

## Results

### Baseline characteristics

A total of 1137 malaria-infected children [6], median age of 5 years, were randomised to AL or DHAPP and SLDPQ/placebo; 239 were G6PD-c.202T (A−) hemizygous males, 45 G6PD-c.202T homozygous females, 119 G6PD A− heterozygous females, and 418 and 202 were G6PD-c.202C normal males and females, respectively (17 unknown status). Some 15% had sickle cell trait and more than half had α-thalassaemia: silent carriers (αα/α −), ~ 43%, and trait (α − /α −), ~ 10%. 1129/1137 (99.3%) were of normal nutritional status. The mean baseline Hb concentration was 10.6 g/dL, ~ 7% (76/1137) had a Hb < 8 g/dL, the asexual parasitaemia geometric mean was 14,599 (range 7–2,172,060)/µL, and 18% had baseline gametocytes. The administered PQ dose ranged from 0.07 to 0.41 mg/kg for a median of 0.21 mg/kg. More details are shown in Additional file 1: Table S1.

### PKPD haemoglobin relationship

**Fig. 2:**
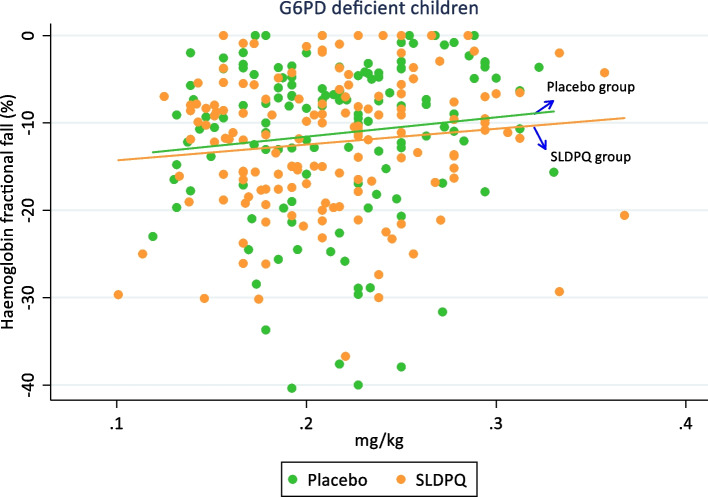
Changes in the fractional fall in haemoglobin as a function of the mg/kg dose of primaquine. The Pearson’s correlation coefficient (*r*) was 0.12 and an increase of 0.1 mg/kg of primaquine predicts a mean decrease in the fractional fall of 1.8 (95% CI: -0.56 to 4.2)%. Results in the placebo group were similar: *r* = 0.16, 2.2 (-1.07 to 5.5)%

In the G6PDd patients, there was substantial overlap in and very similar correlations for the Hb fractional fall between the SLDPQ and placebo recipients (Fig. 2 and Additional file 1: Fig. S3). In the SLDPQ recipients, there was no relationship between Hb fractional fall and the PQ *C*_max_ and AUC (Additional file 1: Fig. S4).

### PKPD gametocyte relationships

**Fig. 3:**
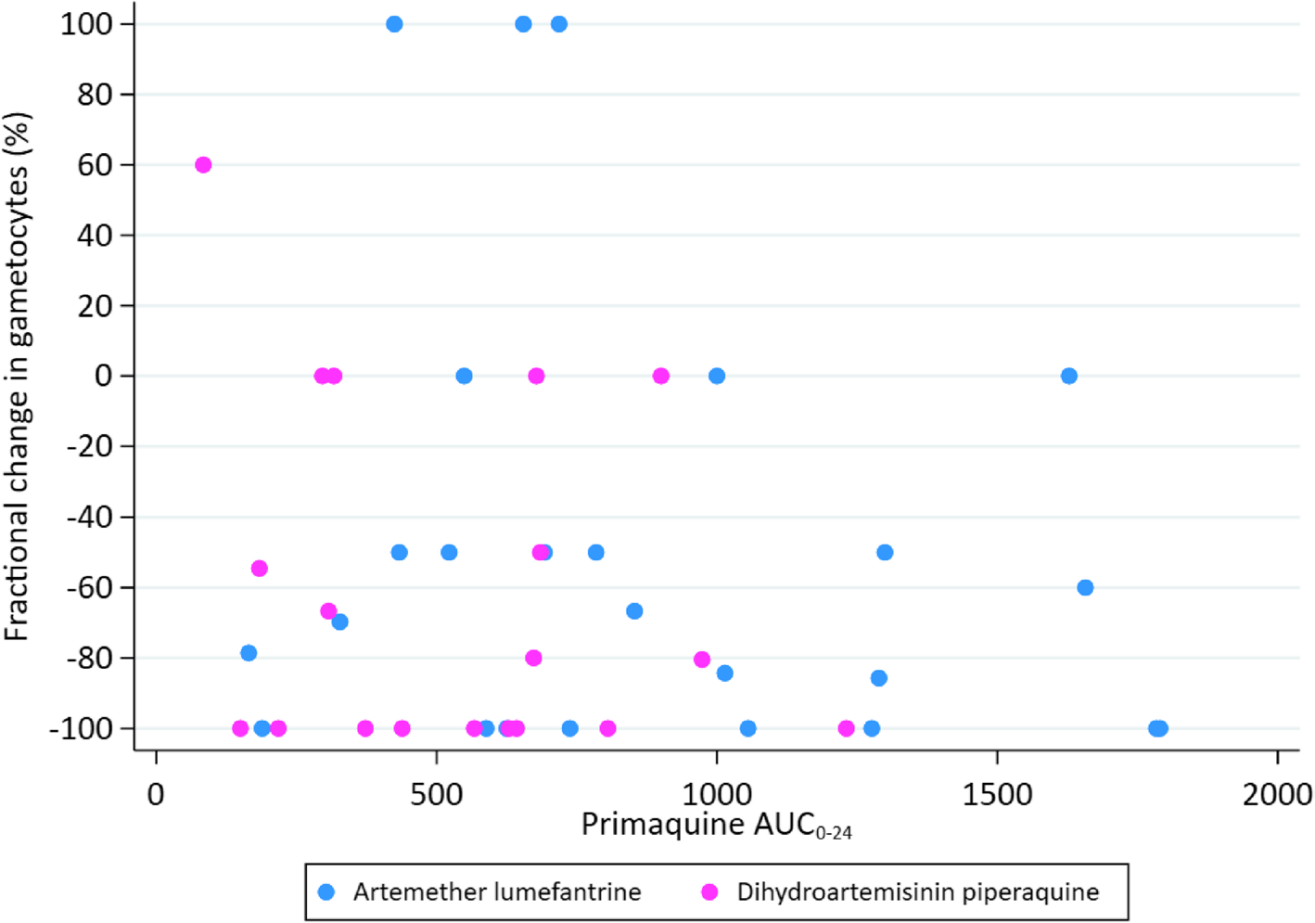
Percent reduction in day 2 gametocytaemia excluding 3 outlying values > 100%, as a function of primaquine exposure [adjusted *R*^2^ (aR.^2^) =  -0.0111, *p* = 0.484, univariate analysis]

There was no relationship between the PQ *C*_max_ (data not shown) and AUC (Fig. 3) and the percent reduction in gametocytaemia on D2. However, children receiving a median dose of ≥ 0.21 mg/kg had significantly lower gametocyte carriage by D3 vs. D7, compared to children receiving < 0.21 mg/kg (Additional file 1: Fig. S5).

### PKPD methaemoglobin relationships

**Fig. 4:**
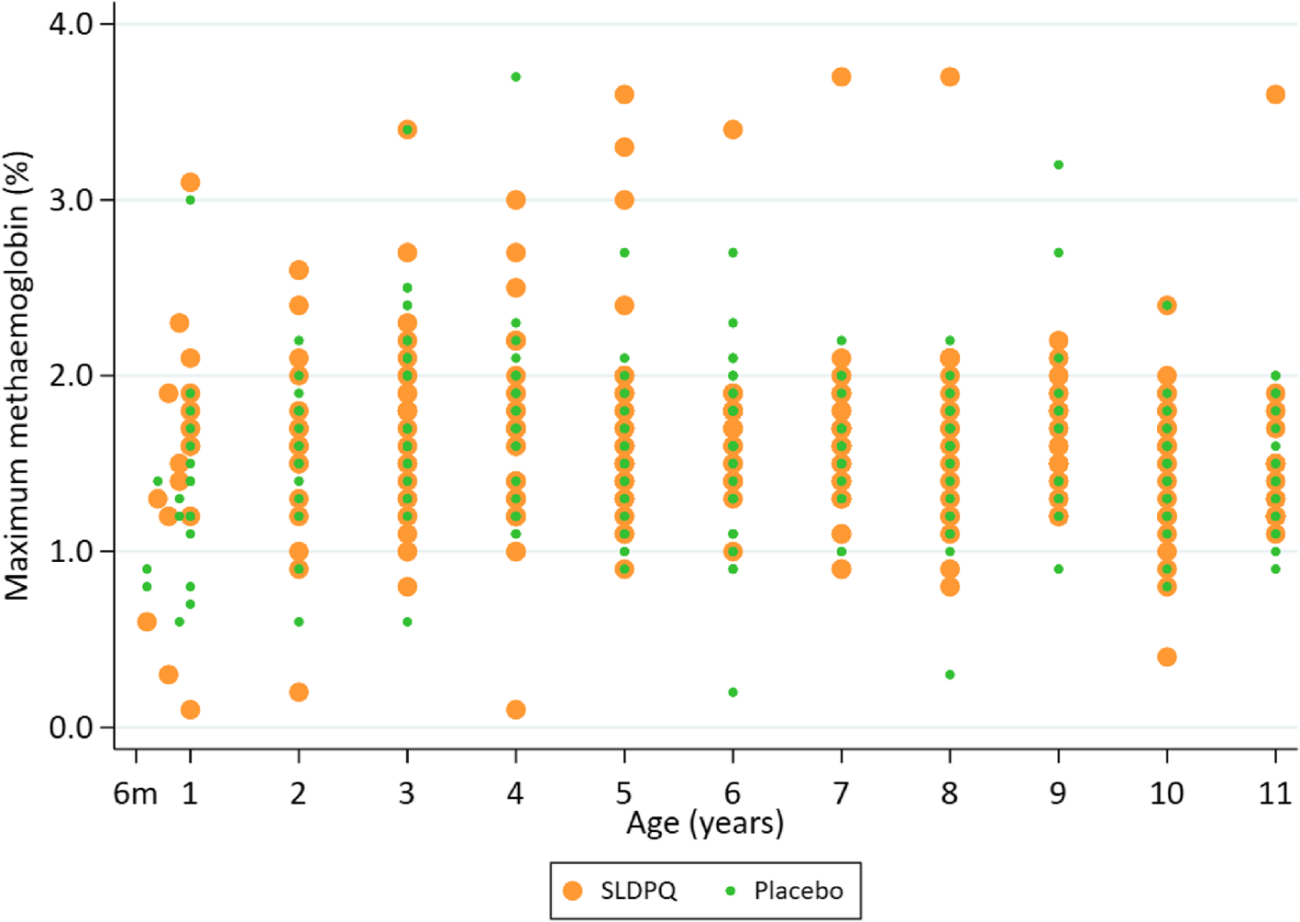
Distribution of peak methaemoglobin values (%) by age in the placebo and single low dose primaquine arms (aR.^2^ =  -0.0015, *p* = 0.419, univariate analysis)

The increases in metHb were modest with much overlap between PQ and placebo. There were no independent associations between the maximum D1 − D3 metHb values and (i) age, (ii) being on SLDPQ/placebo (Fig. 4), and (iii) PQ mg/kg dose, *C*_max_, and AUC (Additional file 1: Fig. S6).

### SLDPQ regimens and predicted exposures

**Fig. 5:**
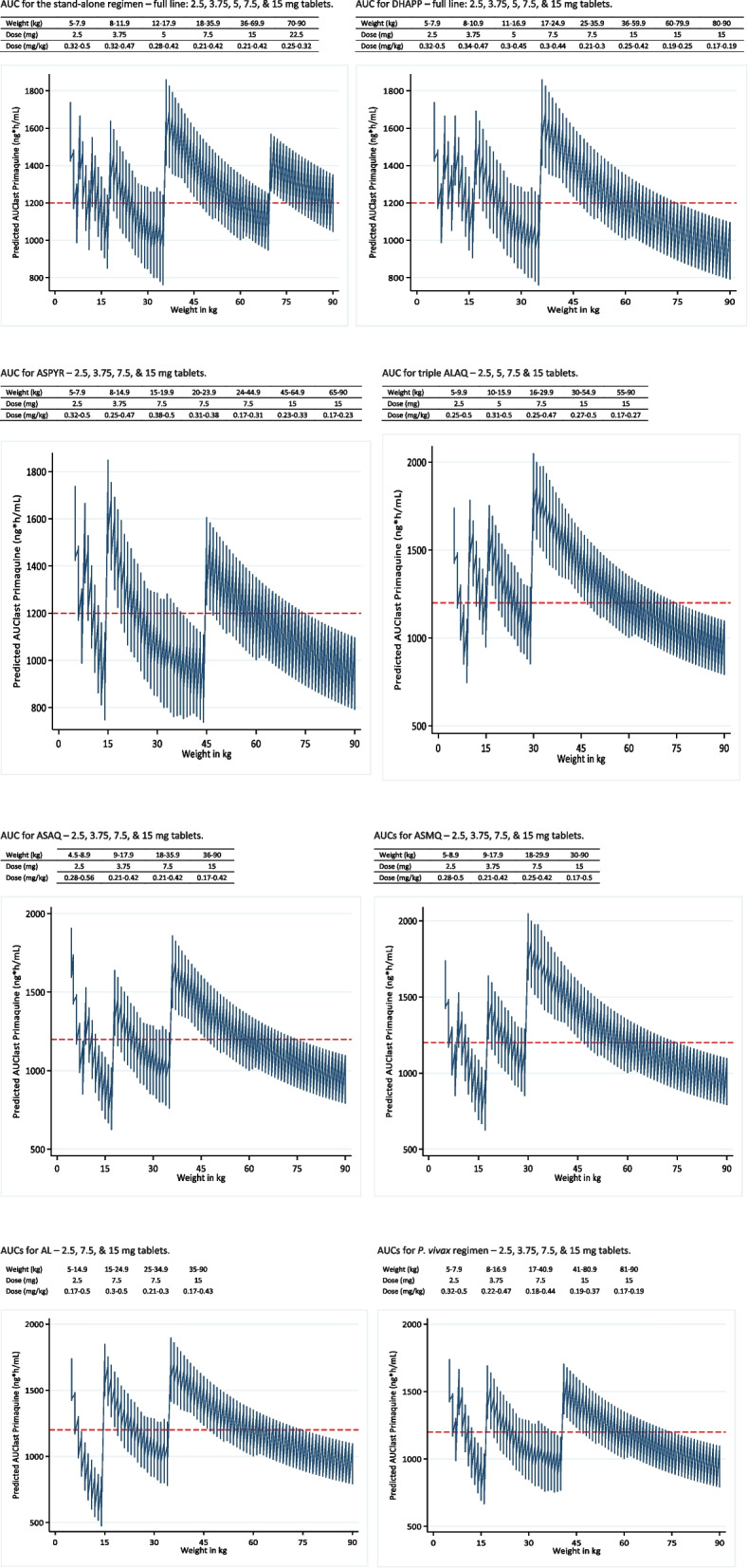
The dosing regimens and their predicted primaquine exposures for the stand-alone regimen and matched regimens for dihydroartemisinin piperaquine (DHAPP), artesunate pyronaridine (ASPYR), ALAQ triple, artesunate amodiaquine (ASAQ), artesunate mefloquine (ASMQ), artemether lumefantrine (AL), and *Plasmodium vivax*. The red line represents the targeted median exposure of 1200 ng*h/mL

The predicted AUCs for the proposed stand-alone, ACT-, triple ACT-, and vivax-matched regimens are shown in Fig. 5. In these regimens, one tablet is equivalent to 1 dose, but in the stand-alone regimen we have used a combination of 7.5 + 15 mg tablets (i.e. 22.5 mg) in the highest weight band. All other regimens, including those with mixed doses of 11.25, 22.5, and 30 mg, are shown in Additional file 1: Fig. S7.

The first dosing band of all regimens uses 2.5 mg of PQ, resulting in maximum doses of 0.56 mg/kg for ASAQ (weight 4.5 kg) and 0.5 mg/kg for all the others. These values are similar to the dose suggested by the allometric equation of 0.47 mg/kg for a 5 kg patient (Additional file 1: Fig. S8). The minimum PQ dose in the first weight band is 0.17 mg/kg in the AL regimen, resulting in an AUC of ~ 500 ng*h/mL compared to 750 − 1000 ng*h/mL minimum values for the other regimens. A 90 kg individual would also receive a minimum of 0.17 mg/kg of PQ with 15 mg, resulting in a predicted minimum exposure of ~ 800 ng*h/mL.

Plots of PQ dose (mg/kg) for selected SLDPQ regimens vs. body weight, overlayed with the allometric line, show greater variation for regimens with fewer dosing bands (Additional file 1: Fig. S8).

The peak median clearance/kg occurred at 18 months. At this age, a child has a median weight of 10 kg with a 5th − 95th centile range of 7.4 − 13.6 kg. If given 3.75 mg of PQ, she/he would receive a median (5th − 95th) dose of 0.375 (0.28 − 0.51) mg/kg, which becomes 0.5 (0.37 − 0.68) mg/kg with a 5 mg tablet. These median mg/kg doses compare to 0.39 mg/kg predicted by the allometric equation. If dosed more tightly by weight, e.g. 8 − 10.9 kg, the resulting mg/kg ranges are 0.34 − 0.47 and 0.46 − 0.63, respectively. Exposures and mg/kg doses for the 3.75 and 5 mg tablets are illustrated in Additional file 1: Figs. S7 and S8.

## Discussion

We have designed SLDPQ regimens for malaria elimination to cover currently used ACTs, a new triple ACT, and, for MCPs who prefer a more straight forward approach, a stand-alone regimen for universal use, and one matched to a proposed vivax regimen for vivax endemic countries.

To derive our regimens, we followed the WHO-recommended target dose of 0.25 mg/kg (15 mg in a 60 kg adult, translating to an AUC of ~ 1200 ng*h/mL [14]), took into account published and newly generated PK data, favouring safety over efficacy, and predefined the tablet strengths with the option of mixing tablets but not using tablet fractions. Although our model was robust and showed a good correlation between observed and predicted AUCs, we extrapolated beyond the data to simulate AUCs outside of the age/weight limits of our patients to make dose recommendations. These AUCs should be considered rough estimates and be interpreted with some caution. Clearly, we need more PQ PK studies in the under-1s, older children, and adults to develop better models. Designing optimal PQ regimens is hampered by the inability to measure the oxidative metabolites that underlie PQ’s mechanisms of action regarding transmitting blocking efficacy and haemolytic toxicity [44, 45]. Thus, dose–response relationships have to be approximated using PQ PK, cognisant that PQ is prodrug that is subject to metabolic polymorphism by CYP 2D6 [45].

MCPs worry about the potential haemolytic toxicity of SLDPQ and this explains partly its limited deployment. Extensive analysis by our group in acutely sick, African children with falciparum malaria [6, 13] has helped establish SLDPQ safety and complements the evidence in Stepniewska et al.’s meta-analysis [15]. With the relatively narrow dose range of 0.07–0.41 mg/kg (IQR 0.17–0.25), there was essentially no relationship between PQ dose and exposure and Hb fractional fall and peak metHb. This is consistent with a small study of healthy G6PDd and normal Malian adults, given higher doses of 0.4, 0.45, and 0.5 mg/kg in whom Hb declines (fractional falls mostly ≤ 10%) and modest metHb increases were unrelated to PQ *C*_max_ and AUC_0-last_ [46]. By contrast, Stepniewska et al. predict an upper 95% CI Hb decrease of 0.34 g/dL/0.1 mg/kg PQ dose increase and a median fall of ~ 3 g/dL for 0.75 mg/kg. These data, data of PQ as mass drug administration [16, 23], and historical data when 0.75 mg/kg was used for transmission blocking [47], all suggest strongly SLDPQ safety across a spectrum of G6PD variants at the 0.25 mg/kg target dose [5, 48].

Most of the proposed regimens use 3.75 mg, resulting in less variability in the younger children and adequate PQ exposures with minimum, predicted AUCs of ~ 600 in ACT regimens (ASAQ and ASMQ), ~ 750 (vivax-matched regimen), and ~ 800–1000 ng*h/mL for the other 3 regimens. The WHO withdrew this tablet strength from prequalification some years back for reasons that remain unclear but they now have the opportunity to reconsider this decision. Moreover, using 5 mg instead of 3.75 mg significantly increases the maximum doses to ~ 0.6 mg/kg and exposures to ~ 2000 ng*h/mL that will likely increase the risk of, particularly, abdominal pain and early vomiting. Background rates of early vomiting in ACT-treated, young children (6–24 months) are ~ 7% for AL and 15% for DHAPP [49], and ~ 2% for AL, ~ 4.5% for ASAQ, and 9% for DHAPP in under-5 children [11]. The lack of a 3.75 mg tablet would also limit options for co-blistering SLDPQ with several ACTs for young children.

All regimens use a 2.5 mg tablet for the first dosing band because this is the lowest, WHO-mandated PQ tablet strength, resulting in maximum mg/kg doses of 0.5 (0.56 for ASAQ) and predicted exposures of ~ 1750 (~ 1900 ASAQ) ng*h/mL. Data on PQ at any dose in very young children who are treated with ACTs are very limited so collecting tolerability data, notably gastrointestinal upset and early vomiting requiring redosing, will be crucial. Our group is in the early stages of developing PQ granules, which are ideally suited for young children. They offer far greater dosing flexibility than fixed dosed tablets and can be given without water, and could, e.g. be dosed at 2 mg in the first weight band to minimise toxicity. Much more development work needs to be done before granules can be registered or, if requested, prequalified by the WHO.

With concrete dosing options for SLDPQ, the question now is where do we go from here? For co-blistering with ACTs, cost and manufacturing practicalities demand that only one PQ tablet is co-blistered but the stand-alone regimen offers the possibility of mixing tablet strengths. The WHO should consider reinstating the 3.75 mg tablet not just for transmission blocking but also for under-1 children with *P. vivax*, if they tolerate poorly the 5 mg dose [41]. Moreover, this would avoid the inconvenience of splitting a 7.5 mg tablet. In considering their next move, many MCPs will to look to the WHO for guidance. The WHO should seize the opportunity to examine the merits of these SLDPQ regimens and incorporate them into the WHO Malaria Guidelines.

Faced with the rapid expansion of de novo artemisinin-resistant *P. falciparum* across eastern Africa [30–34], the WHO needs to act quickly and be bolder in recommending SLDPQ as part of a comprehensive strategy to counter this threat, which, over time, is likely to bring increasing misery, morbidity, and mortality [50, 51]. We cannot wait years for the results of large, cluster-randomised trials to prove that a given strategy impedes the development or spread of artemisinin resistance.

## Conclusions

Some 12 years after the WHO recommended using SLDPQ, we present the first evidenced-based regimens of SLDPQ, offering flexible options for malaria elimination. The proposed doses should be well tolerated across the G6PDd severity spectrum and, with 1 tablet = 1 dose, easily co-blistered and administered. The case for 3.75 mg is compelling and the WHO needs to consider with some urgency reinstating it. More research is needed on PQ granules as a future option for small children.

Time is pressing to counter artemisinin resistance and deployment of SLDPQ needs to be accelerated with the full backing of the WHO.

## Supplementary Information

Additional file 1: Fig. S1 Body weight-normalised clearance of primaquine (14). Fig. S2 Goodness of fit plots for the AUC prediction equation. Fig. S3 The distributions of the nadir haemoglobin (A) and fractional change in haemoglobin (B) from baseline to day of nadir haemoglobin within 14 days in children who received single low dose primaquine or placebo. Fig. S4 Scatter plots of the nadir haemoglobin and fractional change in haemoglobin vs. the maximum primaquine concentration in ng/mL (panels A and B) and primaquine exposure in ng*h/mL (panels C and D). Fig. S5 Gametocyte carriage over time as a function of the mg/kg dose of primaquine. Panel A shows children who received < 0.21 mg/kg and B those received ≥ 0.21 mg/kg. Fig. S6 Scatterplots of the maximum methaemoglobin from days 1 to 3 and the mg/kg dose of primaquine (A), the primaquine *C*_max_ in ng/mL (B), and primaquine exposure in ng*h/mL (C). Fig. S7 Predicted AUCs for single low dose primaquine in ACT-matched and stand-alone regimens. Fig. S8 SLDPQ mg/kg doses of SLDPQ regimens vs. an allometric line. The allometric line formula is adapted from Holford and Anderson (43): primaquine dose = 15 mg × (body weight in kg/60)^0.75^. Table S1 Baseline characteristics of falciparum-infected Ugandan and Congolese children in the safety trail of single low dose primaquine (6). Table S2 Modelled weight for age Table (8). Table S3 Modelled age for weight Table (8).

## Data Availability

We have provided much detailed analysis in this paper. Nevertheless, deidentified individual participant data and relevant supplementary data and documents (e.g., data dictionary, protocol, and participant information sheet) will be available to applicants who provide a sound proposal to the Mahidol Oxford Tropical Medicine Research Unit Data Access Committee (datasharing@tropmedres.ac). A data access agreement will be put in place before sharing.

## Abbreviations

ACT: Artemisinin-based combination therapy
AL: Artemether-lumefantrine
AQ: Amodiaquine
AS: Artesunate or activity score
AUC_0-t_: Area under the drug concentration time curve from 0 to the last PK sample
BW: Body weight
CI: Confidence interval
*C*_max_: Maximum concentration
CYP: Cytochrome
D: Day
DHAPP: Dihydroartemisinin piperaquine
DPP-IMPRIMA: Developing Paediatric Primaquine-Implementing Single Low Dose Primaquine in Africa
IQR: Interquartile range
G6PDd: Glucose-6-phosphate dehydrogenase deficiency
Hb: Haemoglobin
L: Lumefantrine
MCPs: Malaria control programmes
MQ: Mefloquine
PD: Pharmacodynamic
PK: Pharmacokinetics
PQ: Primaquine
PYR: Pyronaridine
SLDPQ: Single low dose primaquine
WHO: World Health Organization
