# Supplementary material for: Single low dose primaquine to block the transmission of Plasmodium falciparum—proposed stand-alone and ACT-adapted regimens
Source: BMC Med. 2025 Jul 1;23:397. doi: 10.1186/s12916-025-04153-4 (PMC12220319; doi:10.1186/s12916-025-04153-4)
Supplement: Supplementary file 1 — Additional file 1: Fig. S1 Body weight-normalised clearance of primaquine (14). Fig. S2 Goodness of fit plots for the AUC prediction equation. Fig. S3 The distributions of the nadir haemoglobin (A) and fractional change in haemoglobin (B) from baseline to day of nadir haemoglobin within 14 days in children who received single low dose primaquine or placebo. Fig. S4 Scatter plots of the nadir haemoglobin and fractional change in haemoglobin vs. the maximum primaquine concentration in ng/mL (panels A and B) and primaquine exposure in ng*h/mL (panels C and D). Fig. S5 Gametocyte carriage over time as a function of the mg/kg dose of primaquine. Panel A shows children who received < 0.21 mg/kg and B those received ≥ 0.21 mg/kg. Fig. S6 Scatterplots of the maximum methaemoglobin from days 1 to 3 and the mg/kg dose of primaquine (A), the primaquine Cmax in ng/mL (B), and primaquine exposure in ng*h/mL (C). Fig. S7 Predicted AUCs for single low dose primaquine in ACT-matched and stand-alone regimens. Fig. S8 SLDPQ mg/kg doses of SLDPQ regimens vs. an allometric line. The allometric line formula is adapted from Holford and Anderson (43): primaquine dose = 15 mg × (body weight in kg/60)0.75. Table S1 Baseline characteristics of falciparum-infected Ugandan and Congolese children in the safety trail of single low dose primaquine (6). Table S2 Modelled weight for age Table (8). Table S3 Modelled age for weight Table (8). [file 12916_2025_4153_MOESM1_ESM.docx]

Contents

[Fig S1. Body weight-normalised clearance of primaquine. 3](#_Toc198308428)

[Fig S2. Goodness of fit plots for the AUC prediction equation. 4](#_Toc198308429)

[Table S1. Baseline characteristics of falciparum-infected Ugandan and Congolese children in the safety trial of single low dose primaquine. 5](#_Toc198308430)

[Fig S3. The distributions of the nadir haemoglobin (A) and fractional change in haemoglobin (B) from baseline to day of nadir haemoglobin within 14 days in children who received single low dose primaquine or placebo. 7](#_Toc198308431)

[Fig S4. Scatter plots of the nadir haemoglobin and fractional change in haemoglobin vs. the maximum primaquine concentration in ng/mL (panels A & B) and primaquine exposure in ng*h/mL (Panels C & D). 8](#_Toc198308432)

[Fig S5. Gametocyte carriage over time as a function of the mg/kg dose of primaquine. Panel A shows children who received <0.21 mg/kg and B those received ≥0.21 mg/kg. 9](#_Toc198308433)

[Fig S6. Scatterplots of the maximum methaemoglobin from Days 1 to 3 and the mg/kg dose of primaquine (A), the primaquine *C*_max_ in ng/mL (B), and primaquine exposure in ng*h/mL (C). 10](#_Toc198308434)

[Fig S7. Predicted AUCs for SLDPQ in stand-alone, all ACT-matched & vivax-matched regimens. 11](#_Toc198308435)

[AUC stand-alone DPP-IMPRIMA regimen. 11](#_Toc198308436)

[Primaquine AUC – full line with 22.5 mg in the last dosing band. 11](#_Toc198308437)

[Primaquine AUC – 5 mg replaces 3.75 mg in band 2. 11](#_Toc198308438)

[Primaquine AUC – full line using 15 mg in the last weight band. 12](#_Toc198308439)

[AUC DHAPP. 13](#_Toc198308440)

[Primaquine AUC for DHAPP – full line. 13](#_Toc198308441)

[Primaquine AUC for DHAPP – 5 mg replaces 3.75 mg in band 2. 13](#_Toc198308442)

[Primaquine AUC for DHAPP – using 30 mg in band 8. 14](#_Toc198308443)

[AUC ASPYR. 15](#_Toc198308444)

[Primaquine AUC for ASPYR – full line. 15](#_Toc198308445)

[Primaquine AUC for ASPYR – 5 mg replaces 3.75 mg in band 2. 15](#_Toc198308446)

[Primaquine AUC for ASPYR – 7.5 mg replaces 5 mg in band 3. 16](#_Toc198308447)

[Primaquine AUC for ASPYR – using 11.25 mg in band 5. 16](#_Toc198308448)

[AUC ALAQ triple ACT. 17](#_Toc198308449)

[Primaquine AUC for ALAQ – using 3.75 mg. 17](#_Toc198308450)

[Primaquine AUC for ALAQ – 5 mg replaces 3.75 mg in band 2. 17](#_Toc198308451)

[Primaquine AUC for ALAQ – using 11.25 mg in band 4. 18](#_Toc198308452)

[Primaquine AUC for ALAQ – using 22.5 mg in band 5. 18](#_Toc198308453)

[AUC ASAQ. 19](#_Toc198308454)

[Primaquine AUC for ASAQ – using 3.75 mg. 19](#_Toc198308455)

[Primaquine AUC for ASAQ – 5 mg replaces 3.75 mg in band 2. 19](#_Toc198308456)

[AUC ASMQ 20](#_Toc198308457)

[Primaquine AUC for ASMQ – using 3.75 mg. 20](#_Toc198308458)

[Primaquine AUC for ASMQ – 5 mg replaces 3.75 mg in band 2. 20](#_Toc198308459)

[AUC AL. 21](#_Toc198308460)

[Primaquine AUCs for two SLDPQ options: 5 or 7.5 mg in band 2. 21](#_Toc198308461)

[AUC for SLDPQ matched to a *P. vivax* radical cure regimen. 22](#_Toc198308462)

[Primaquine AUC vivax-matched – using 3.75 mg. 22](#_Toc198308463)

[Primaquine AUC vivax-matched – 5 mg replaces 3.75 mg in band 2. 22](#_Toc198308464)

[Primaquine AUCs vivax-matched – using 22.5 & 30 mg in the last weight band. 23](#_Toc198308465)

[Fig S8. Mg/kg doses of SLDPQ regimens vs. an allometric line. 24](#_Toc198308466)

[Stand-alone DPP-IMPRIMA regimen – full line. 24](#_Toc198308467)

[Stand-alone DPP-IMPRIMA regimen mg/kg – 5 mg replaces 3.75 mg. 25](#_Toc198308468)

[DHAPP – full line. 26](#_Toc198308469)

[DHAPP – 5 mg replaces 3.75 mg. 27](#_Toc198308470)

[ASPYR – full line. 28](#_Toc198308471)

[ASPYR – 5 mg replaces 3.5 mg. 29](#_Toc198308472)

[ASPYR – 7.5 mg replaces 5 mg in band 3. 30](#_Toc198308473)

[Triple ALAQ – using 3.75 mg. 31](#_Toc198308474)

[Triple ALAQ – 5 mg replaces 3.75 mg. 32](#_Toc198308475)

[ASAQ – using 3.75 mg. 33](#_Toc198308476)

[ASAQ – 5 mg replaces 3.75 mg. 34](#_Toc198308477)

[ASMQ – using 3.75 mg. 35](#_Toc198308478)

[ASMQ – 5 mg replaces 3.75 mg. 36](#_Toc198308479)

[AL – 5 mg in the second dosing band. 37](#_Toc198308480)

[AL – 7.5 mg in the second dosing band. 38](#_Toc198308481)

[Vivax-matched regimen – using 3.75 mg. 39](#_Toc198308482)

[Vivax-matched regimen – 5 mg replaces 3.75 mg. 40](#_Toc198308483)

[Table S1. Modelled weight for age table. 41](#_Toc198308484)

[Table S2. Age weight table. 43](#_Toc198308485)

# Fig S1. Body weight-normalised clearance of primaquine.

# Fig S2. Goodness of fit plots for the AUC prediction equation.


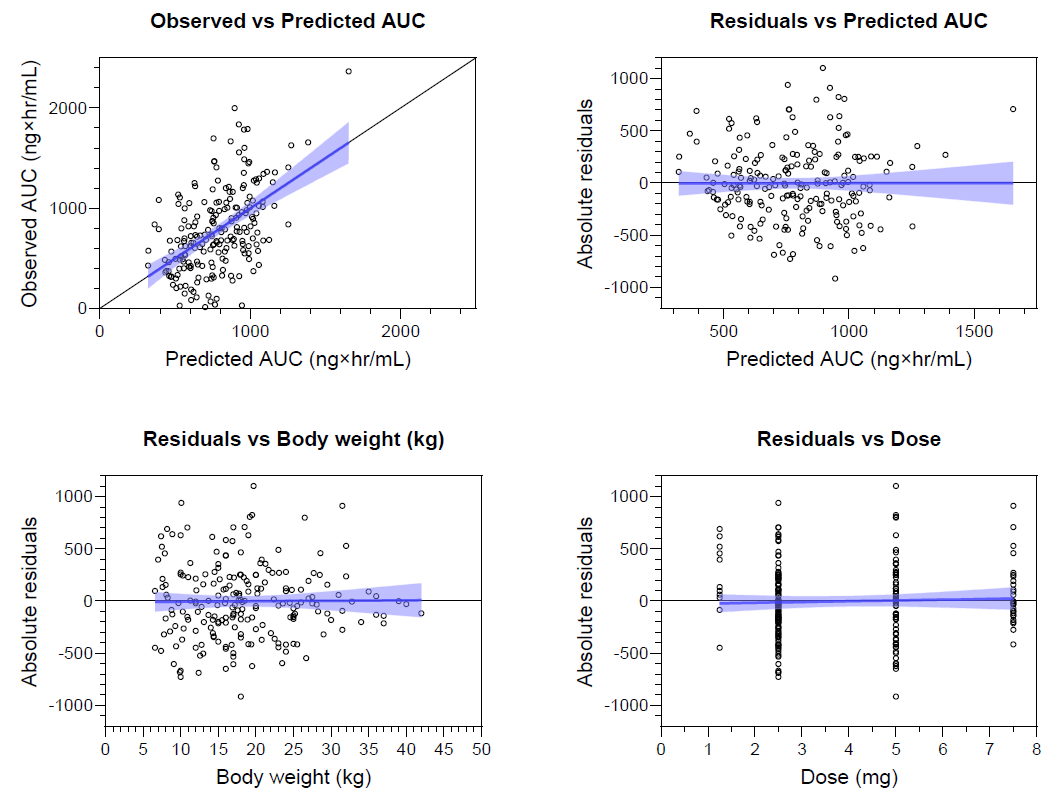


# Table S1. Baseline characteristics of falciparum-infected Ugandan and Congolese children in the safety trial of single low dose primaquine.


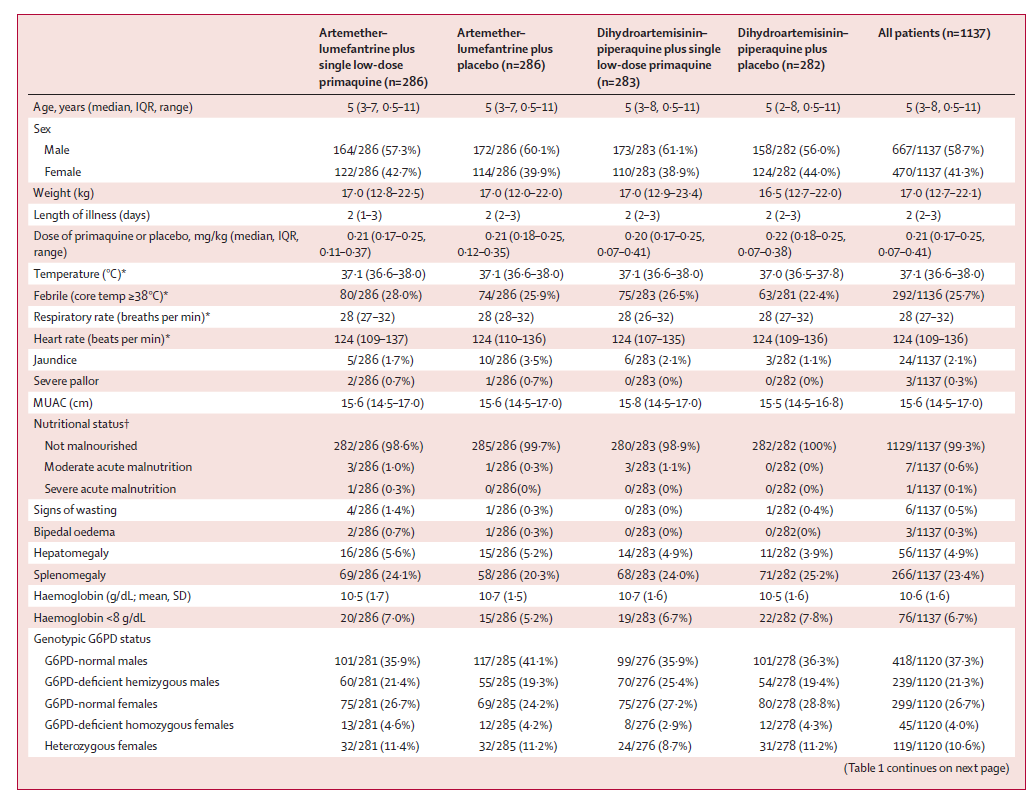


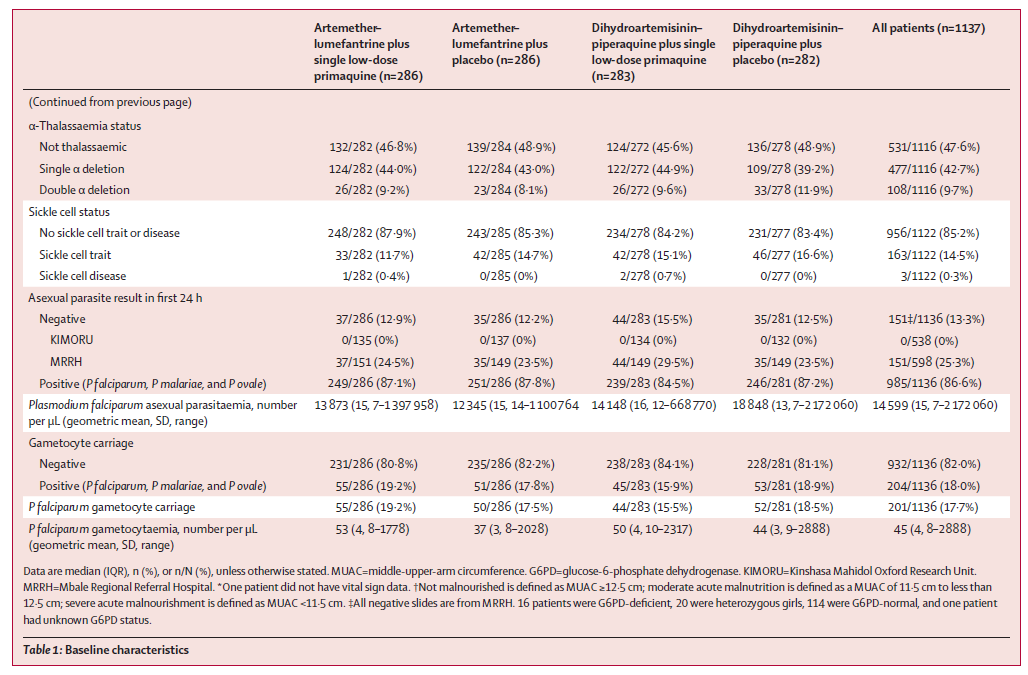


# Fig S3. The distributions of the nadir haemoglobin (A) and fractional change in haemoglobin (B) from baseline to day of nadir haemoglobin within 14 days in children who received single low dose primaquine or placebo.

| A  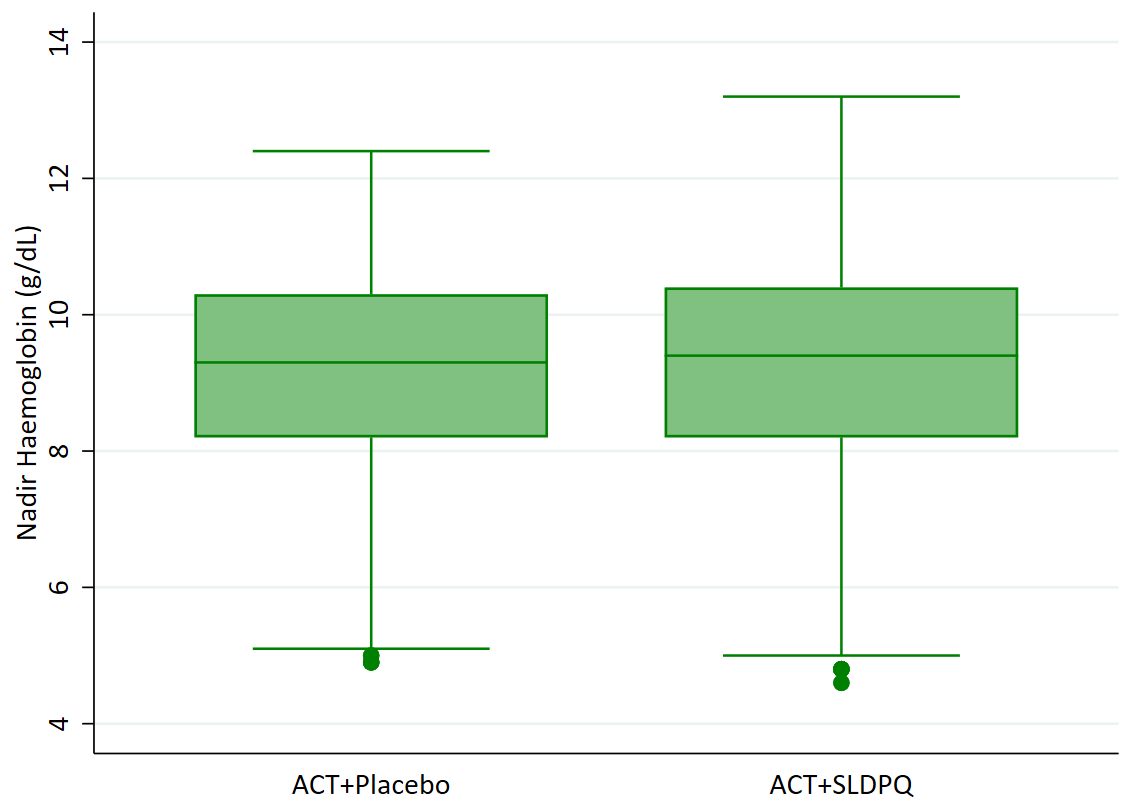 | B  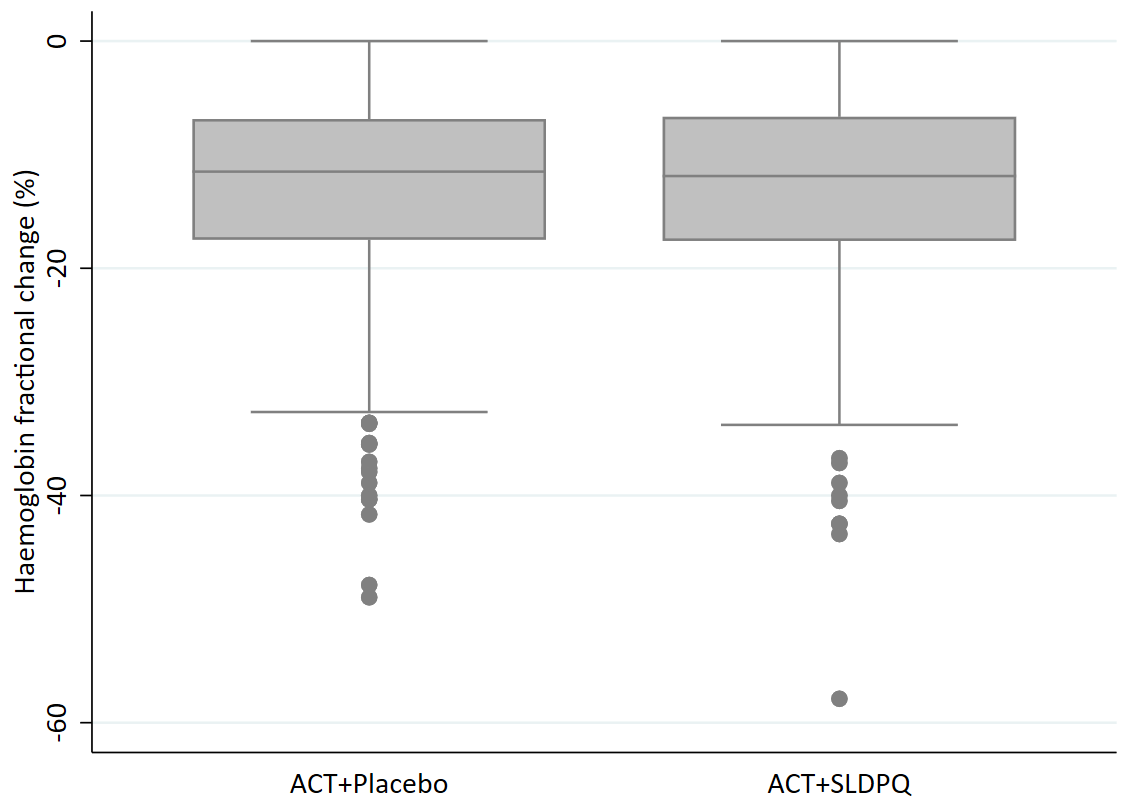 |
| --- | --- |

# Fig S4. Scatter plots of the nadir haemoglobin and fractional change in haemoglobin vs. the maximum primaquine concentration in ng/mL (panels A & B) and primaquine exposure in ng*h/mL (Panels C & D).

| A | B |
| --- | --- |
| 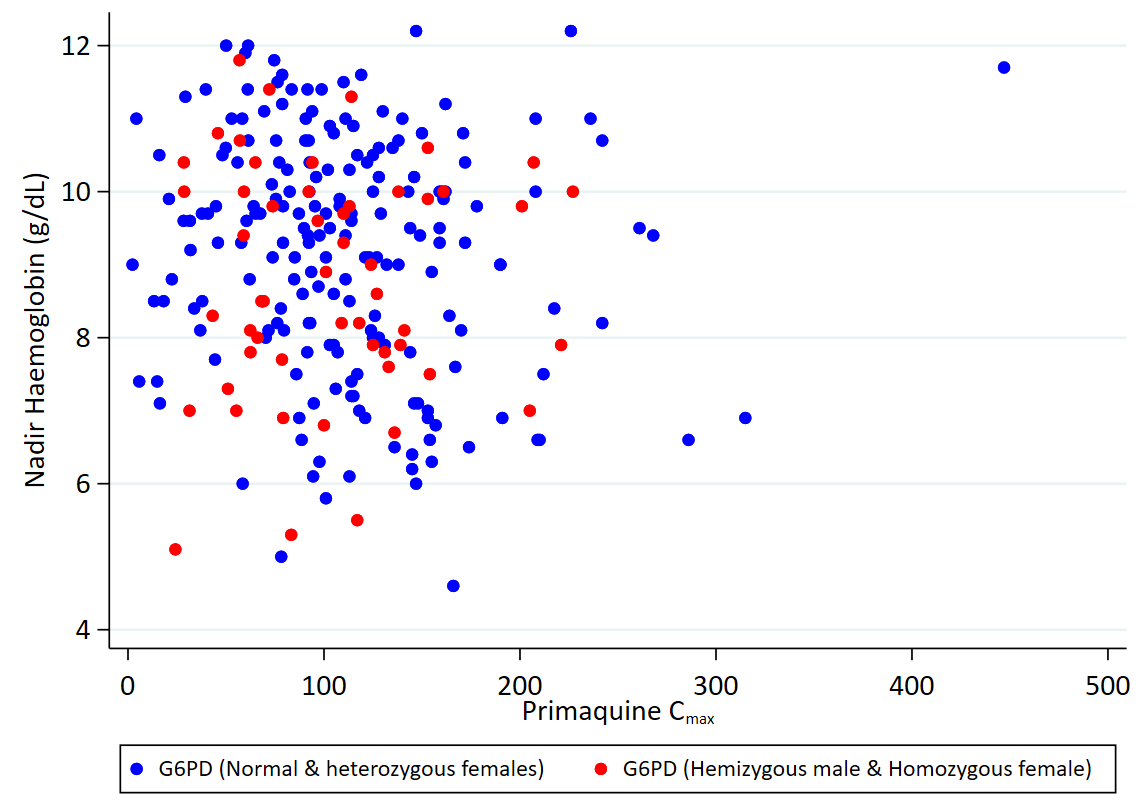 | 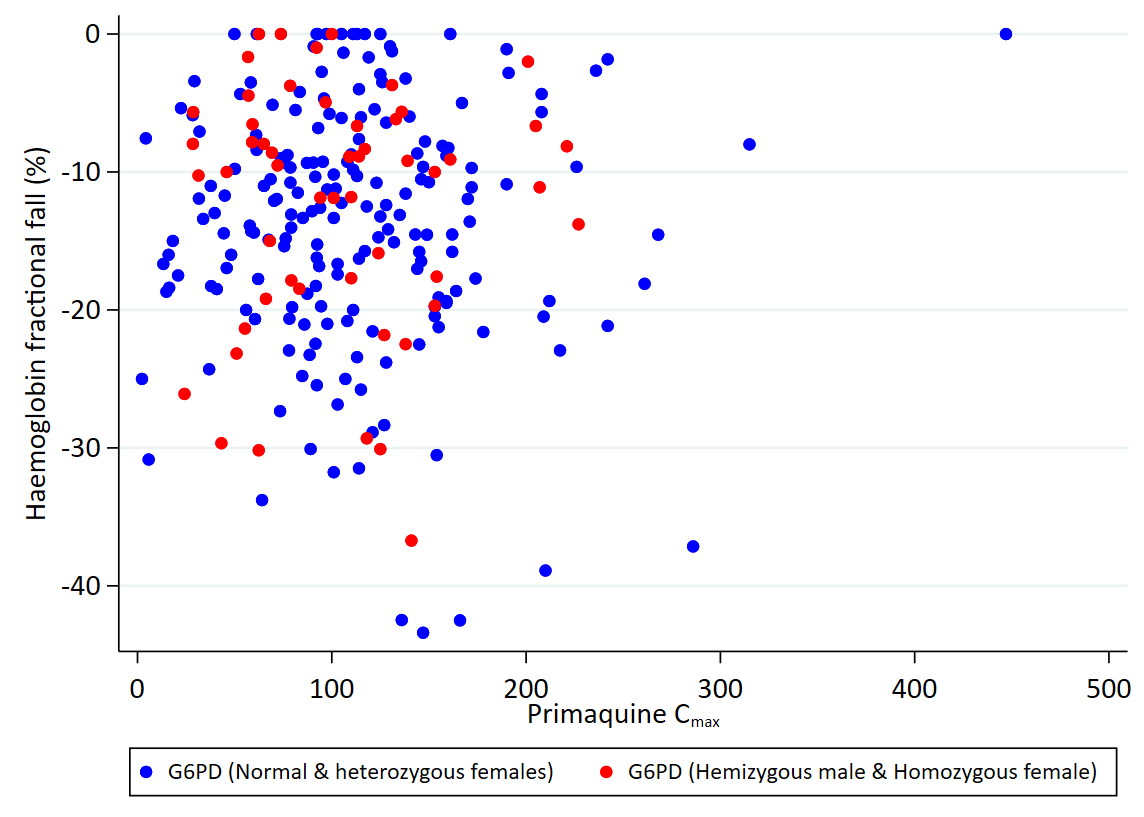 |
| C | D |
| 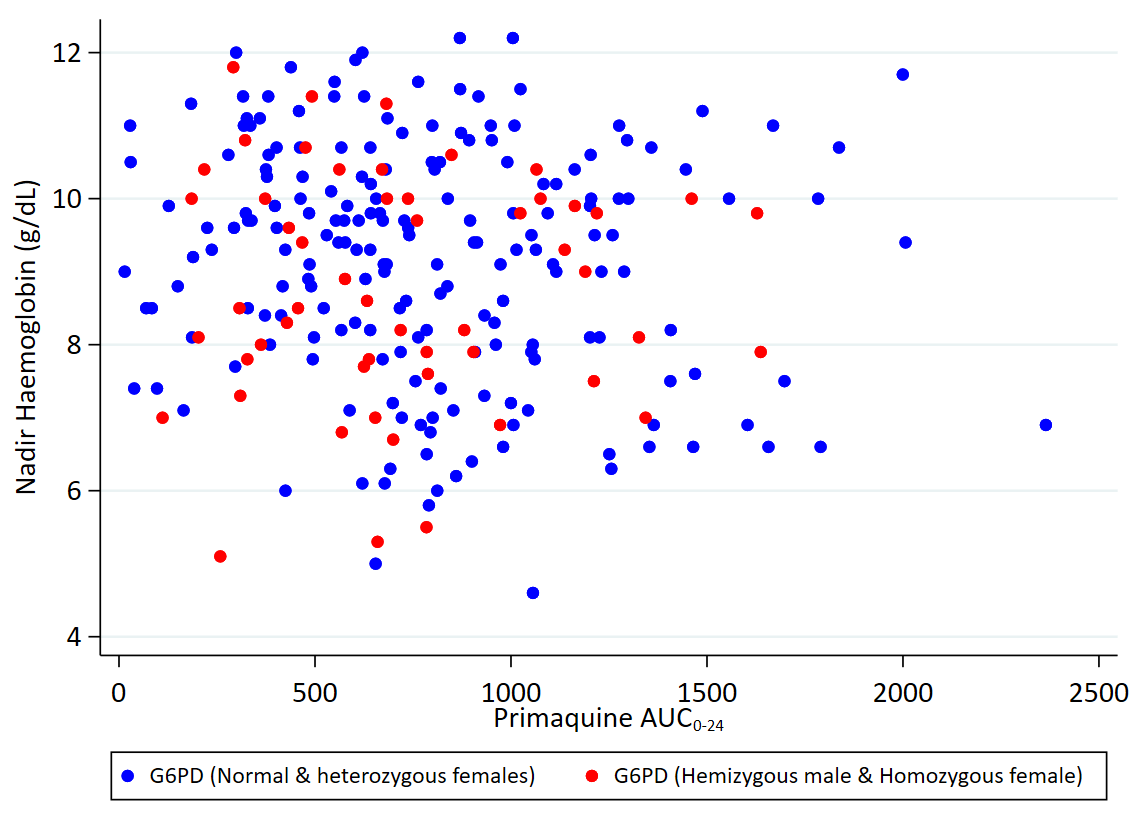 | 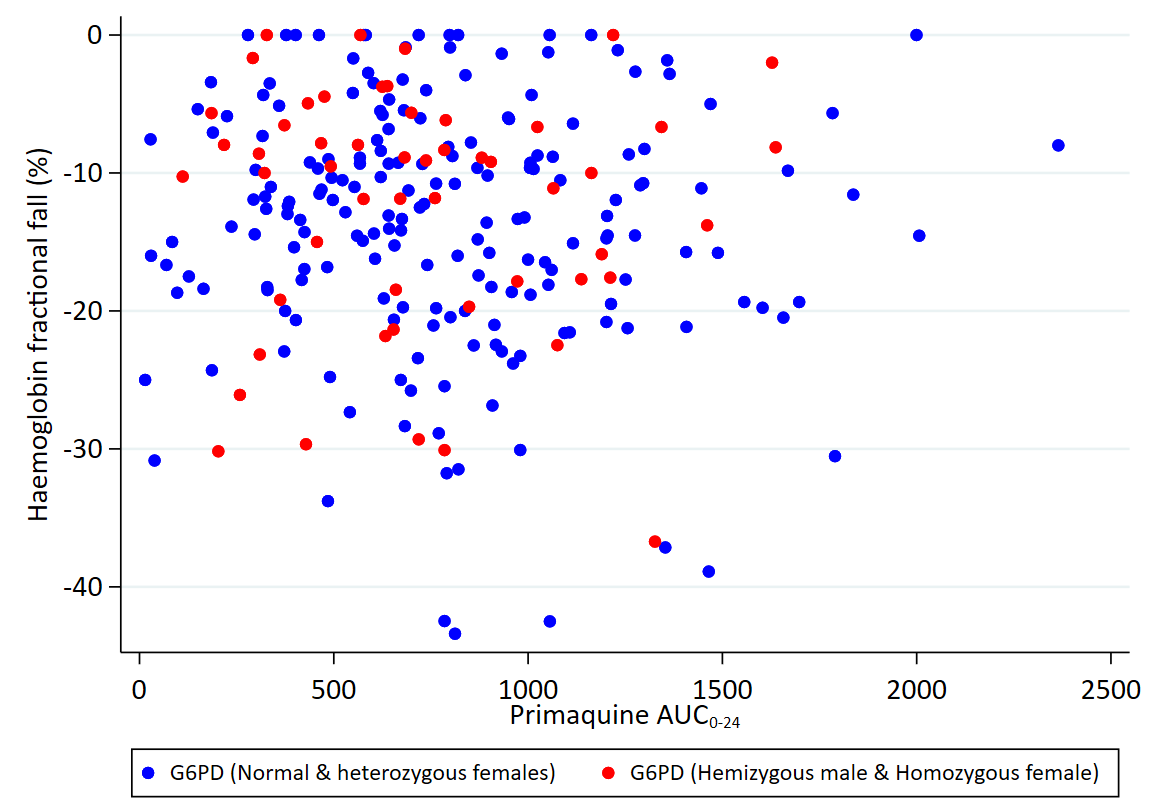 |

# Fig S5. Gametocyte carriage over time as a function of the mg/kg dose of primaquine. Panel A shows children who received <0.21 mg/kg and B those received ≥0.21 mg/kg.

| A | B |
| --- | --- |
| 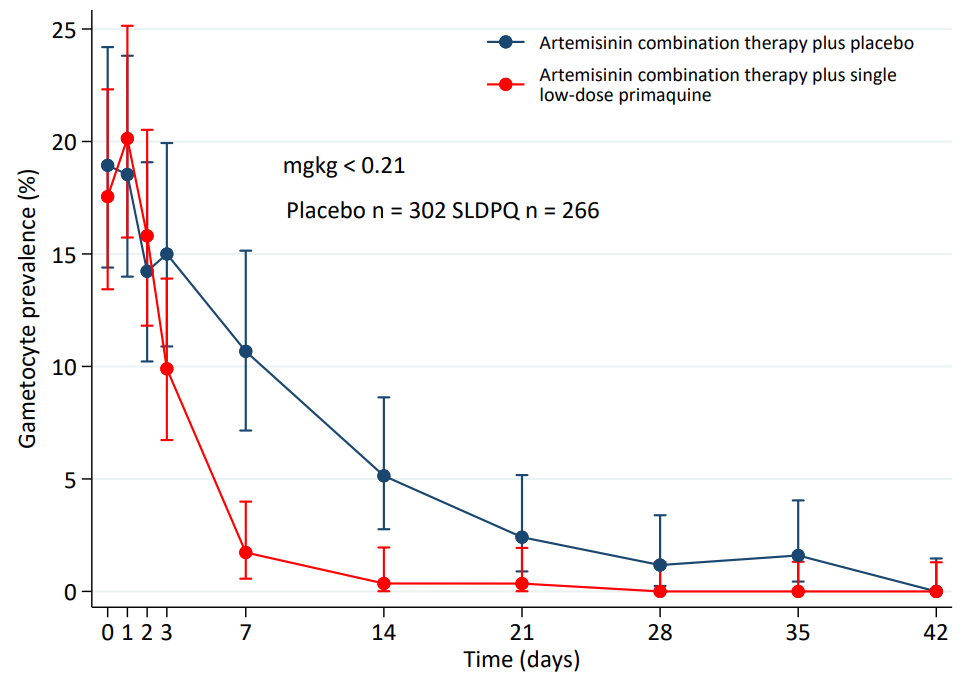 | 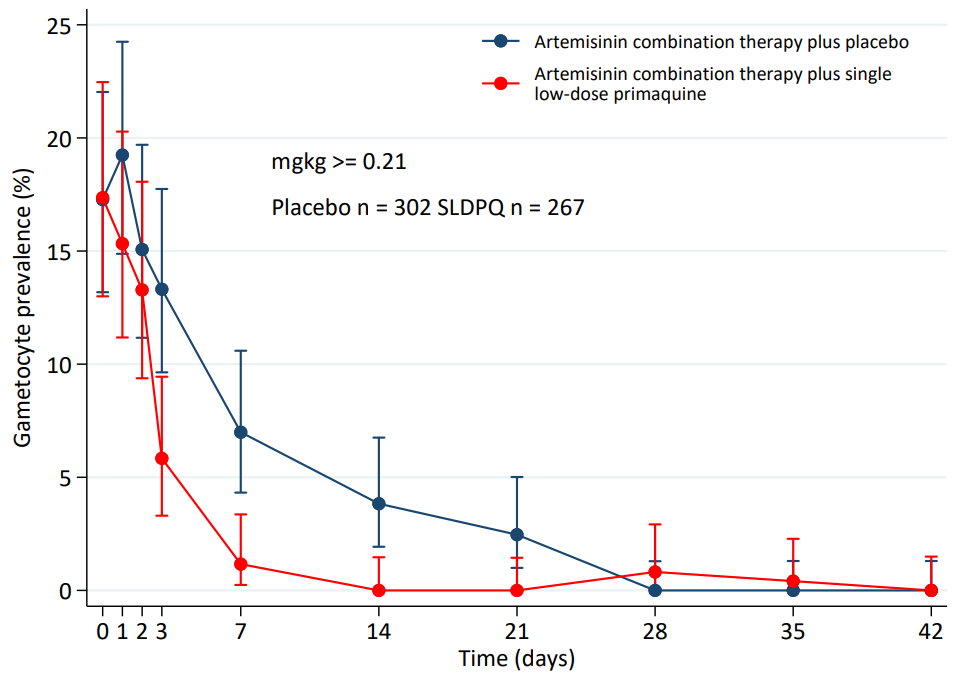 |

# Fig S6. Scatterplots of the maximum methaemoglobin from Days 1 to 3 and the mg/kg dose of primaquine (A), the primaquine *C*_max_ in ng/mL (B), and primaquine exposure in ng*h/mL (C).

| A    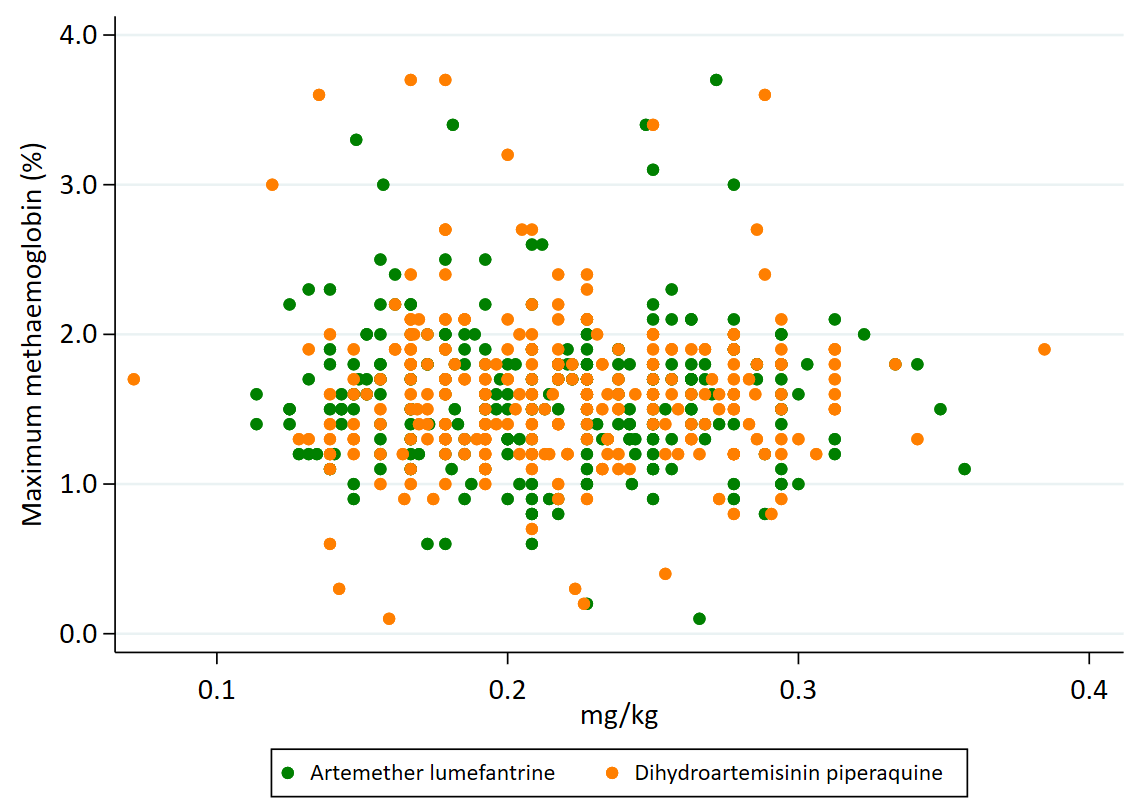 |
| --- |
| B  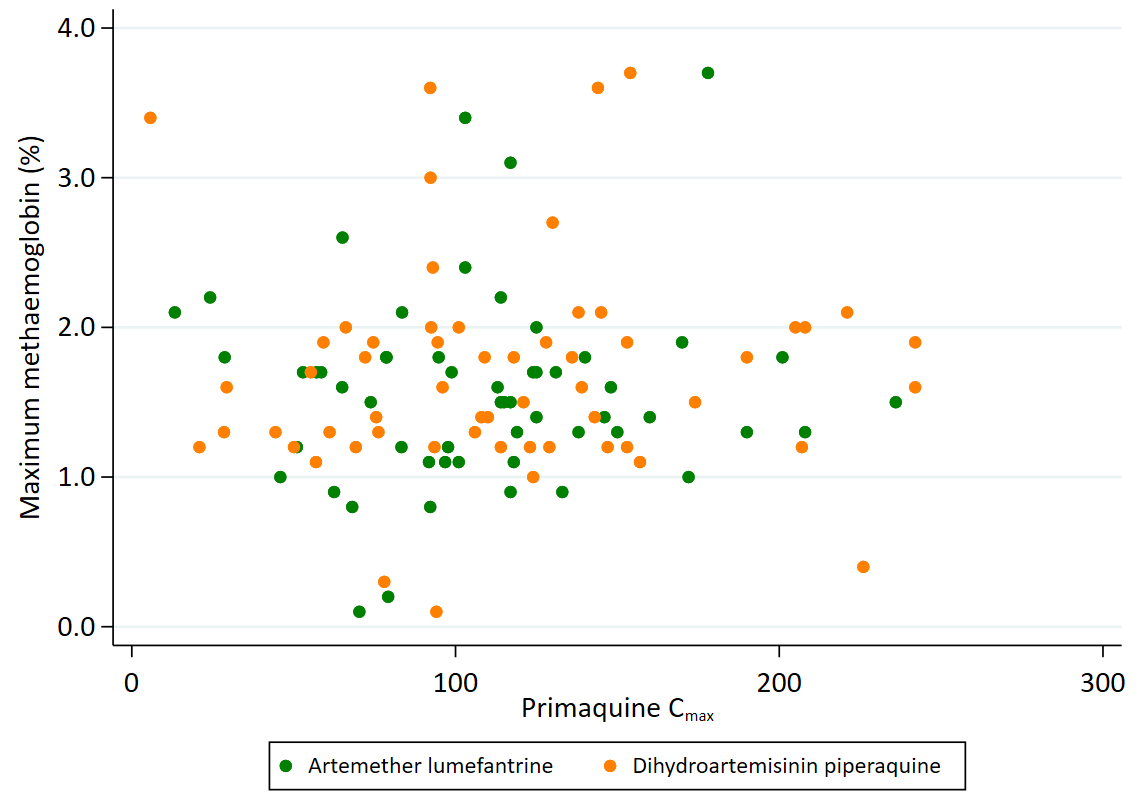 |
| C  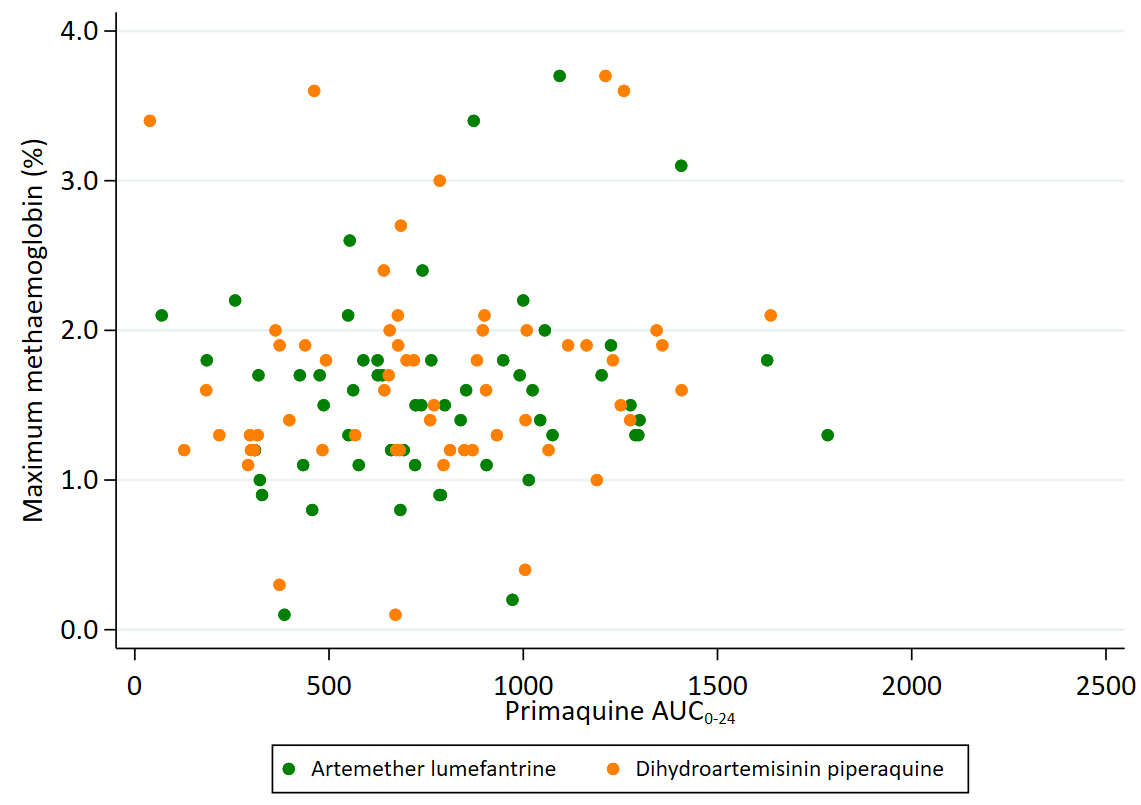 |

# Fig S7. Predicted AUCs for SLDPQ in stand-alone, all ACT-matched & vivax-matched regimens.

# AUC stand-alone DPP-IMPRIMA regimen.

## Primaquine AUC – full line with 22.5 mg in the last dosing band.

| 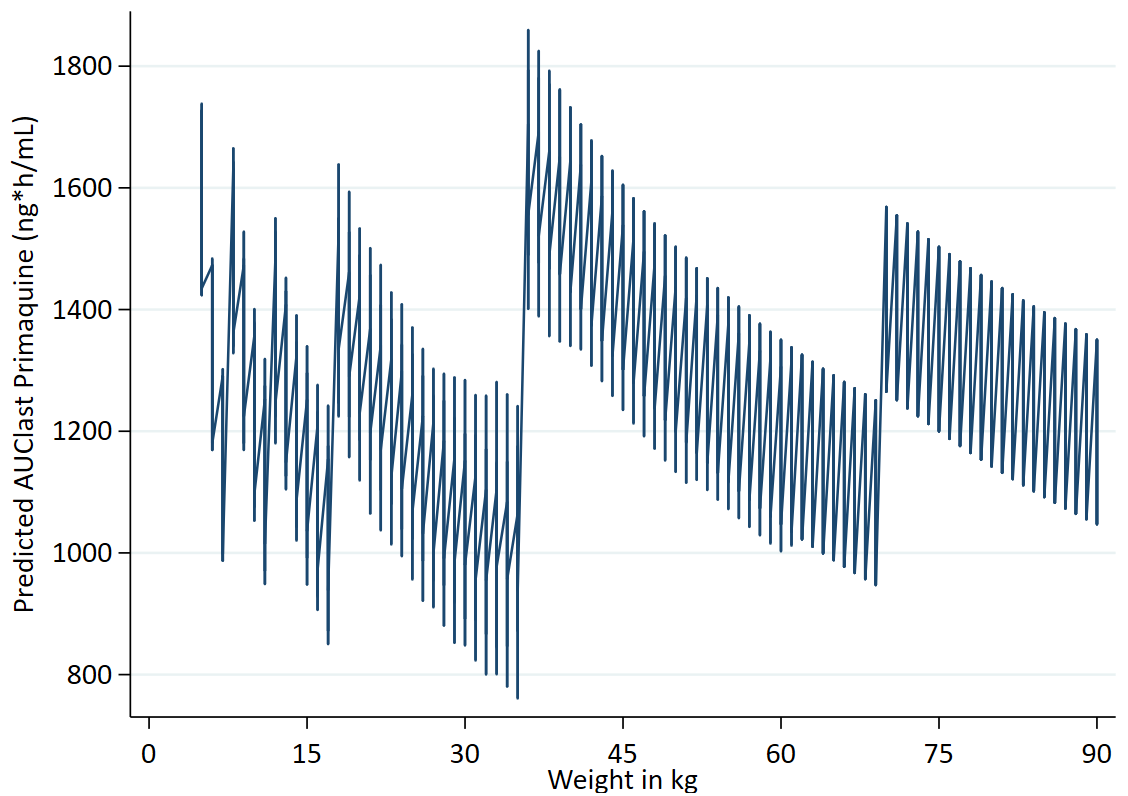 | \| **Weight** \| **Dose** \| **mg/kg** \| \| \| --- \| --- \| --- \| --- \| \| 5 to 7.9 \| 2.50 \| 0.32 \| 0.50 \| \| 8 to 11.9 \| 3.75 \| 0.32 \| 0.47 \| \| 12 to 17.9 \| 5 \| 0.28 \| 0.42 \| \| 18 to 35.9 \| 7.5 \| 0.21 \| 0.42 \| \| 36 to 69.9 \| 15 \| 0.21 \| 0.42 \| \| 70 to 90 \| 22.5 \| 0.25 \| 0.32 \| |
| --- | --- | --- | --- | --- | --- | --- | --- | --- | --- | --- | --- | --- | --- | --- | --- | --- | --- | --- | --- | --- | --- | --- | --- | --- | --- | --- | --- | --- | --- |

## Primaquine AUC – 5 mg replaces 3.75 mg in band 2.

| **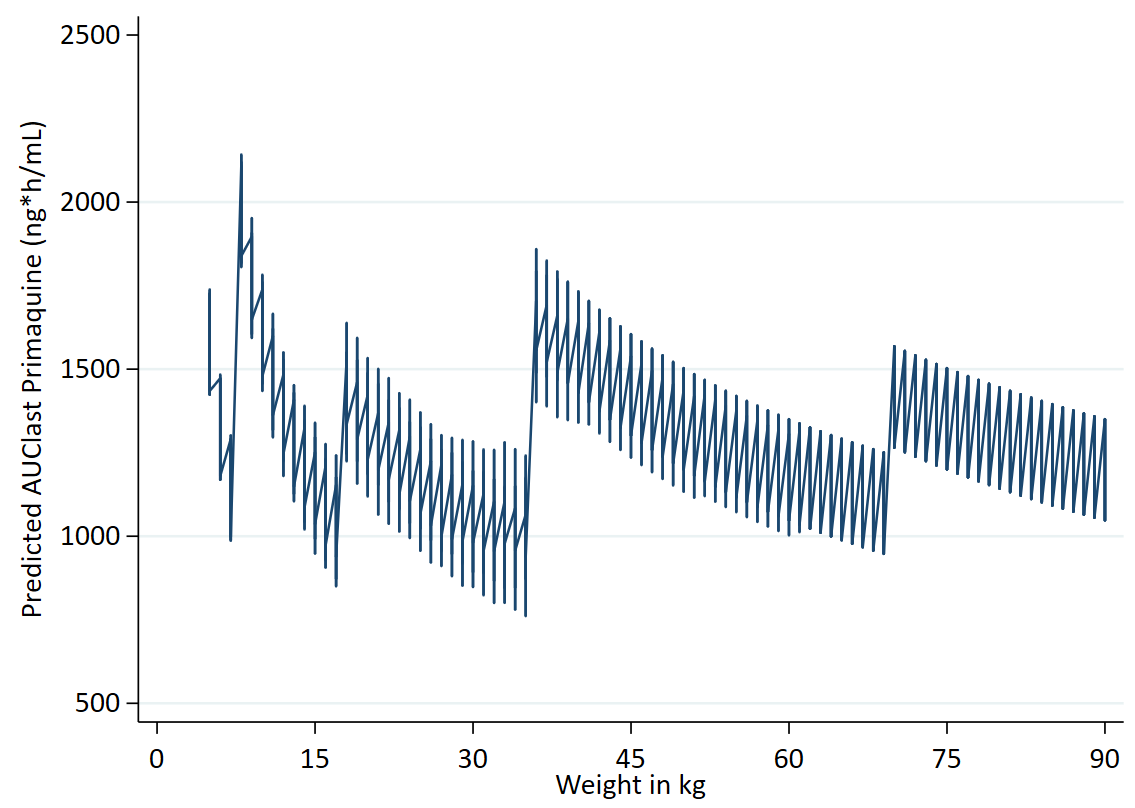** | \| **Weight** \| **Dose** \| **mg/kg** \| \| \| --- \| --- \| --- \| --- \| \| 5 to 7.9 \| 2.50 \| 0.32 \| 0.50 \| \| 8 to 11.9 \| 5 \| 0.42 \| 0.63 \| \| 12 to 17.9 \| 5 \| 0.28 \| 0.42 \| \| 18 to 35.9 \| 7.5 \| 0.21 \| 0.42 \| \| 36 to 69.9 \| 15 \| 0.21 \| 0.42 \| \| 70 to 90 \| 22.5 \| 0.25 \| 0.32 \| |
| --- | --- | --- | --- | --- | --- | --- | --- | --- | --- | --- | --- | --- | --- | --- | --- | --- | --- | --- | --- | --- | --- | --- | --- | --- | --- | --- | --- | --- | --- |

## Primaquine AUC – full line using 15 mg in the last weight band.

| **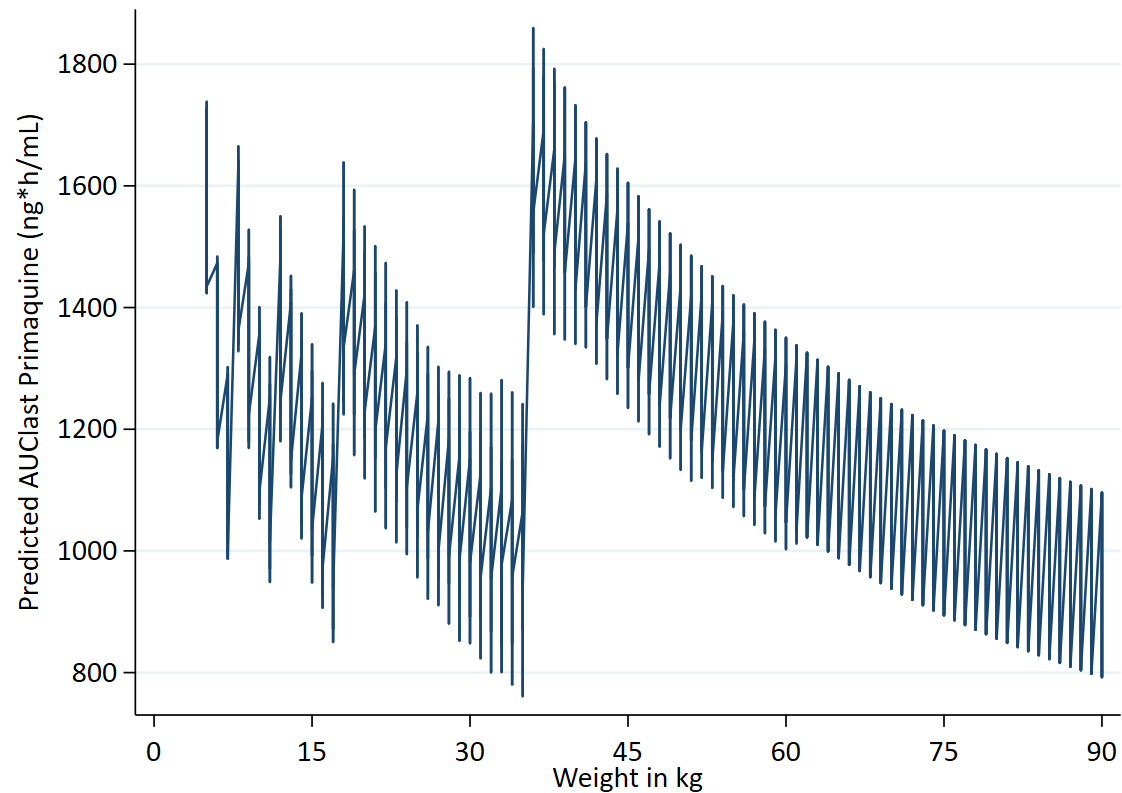** | \| **Weight** \| **Dose** \| **mg/kg** \| \| \| --- \| --- \| --- \| --- \| \| 5 to 7.9 \| 2.5 \| 0.32 \| 0.5 \| \| 8 to 11.9 \| 3.75 \| 0.32 \| 0.47 \| \| 12 to 17.9 \| 5 \| 0.28 \| 0.42 \| \| 18 to 35.9 \| 7.5 \| 0.21 \| 0.42 \| \| 36 to 69.9 \| 15 \| 0.21 \| 0.42 \| \| 70 to 90 \| 15 \| 0.21 \| 0.17 \| |
| --- | --- | --- | --- | --- | --- | --- | --- | --- | --- | --- | --- | --- | --- | --- | --- | --- | --- | --- | --- | --- | --- | --- | --- | --- | --- | --- | --- | --- | --- |

# AUC DHAPP.

## Primaquine AUC for DHAPP – full line.

| **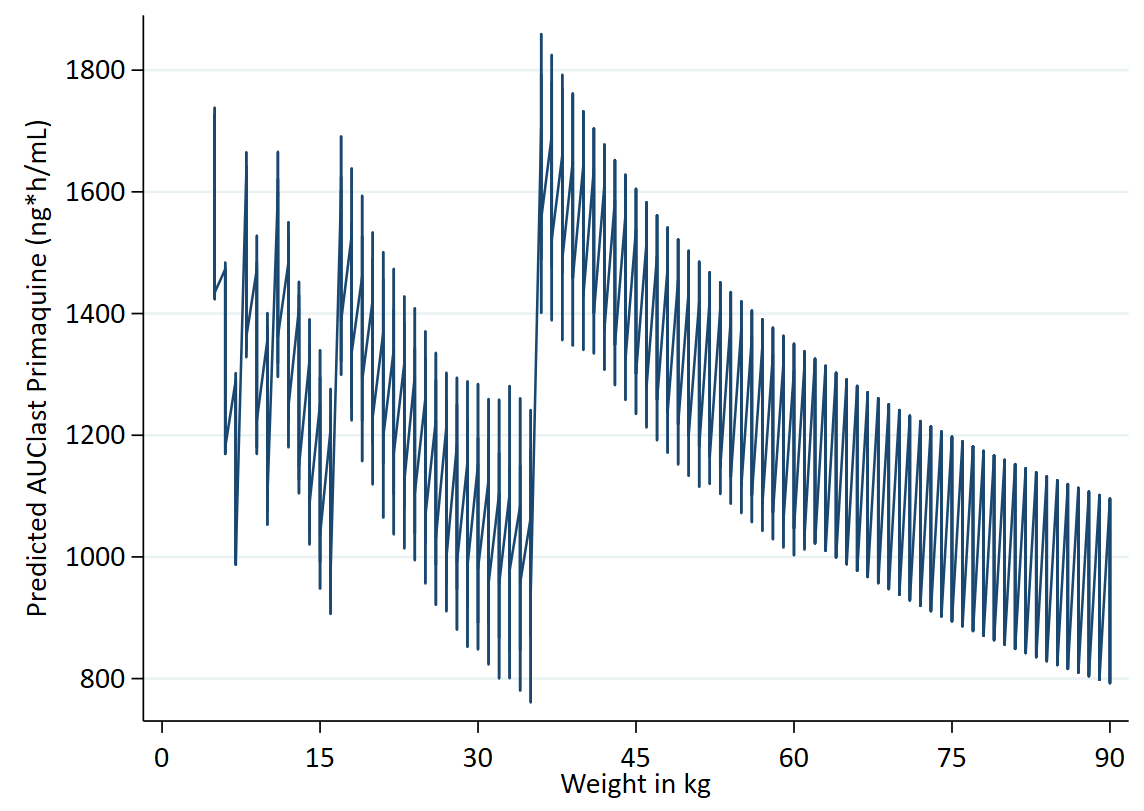** | \| **Weight** \| **Dose** \| **mg/kg** \| \| \| --- \| --- \| --- \| --- \| \| 5 to 7.9 \| 2.5 \| 0.32 \| 0.50 \| \| 8 to 10.9 \| 3.75 \| 0.34 \| 0.47 \| \| 11 to 16.9 \| 5 \| 0.30 \| 0.45 \| \| 17 to 24.9 \| 7.5 \| 0.30 \| 0.44 \| \| 25 to 35.9 \| 7.5 \| 0.21 \| 0.30 \| \| 36 to 59.9 \| 15 \| 0.25 \| 0.42 \| \| 60 to 79.9 \| 15 \| 0.19 \| 0.25 \| \| 80 to 90 \| 15 \| 0.17 \| 0.19 \| |
| --- | --- | --- | --- | --- | --- | --- | --- | --- | --- | --- | --- | --- | --- | --- | --- | --- | --- | --- | --- | --- | --- | --- | --- | --- | --- | --- | --- | --- | --- | --- | --- | --- | --- | --- | --- | --- | --- |

## Primaquine AUC for DHAPP – 5 mg replaces 3.75 mg in band 2.

| **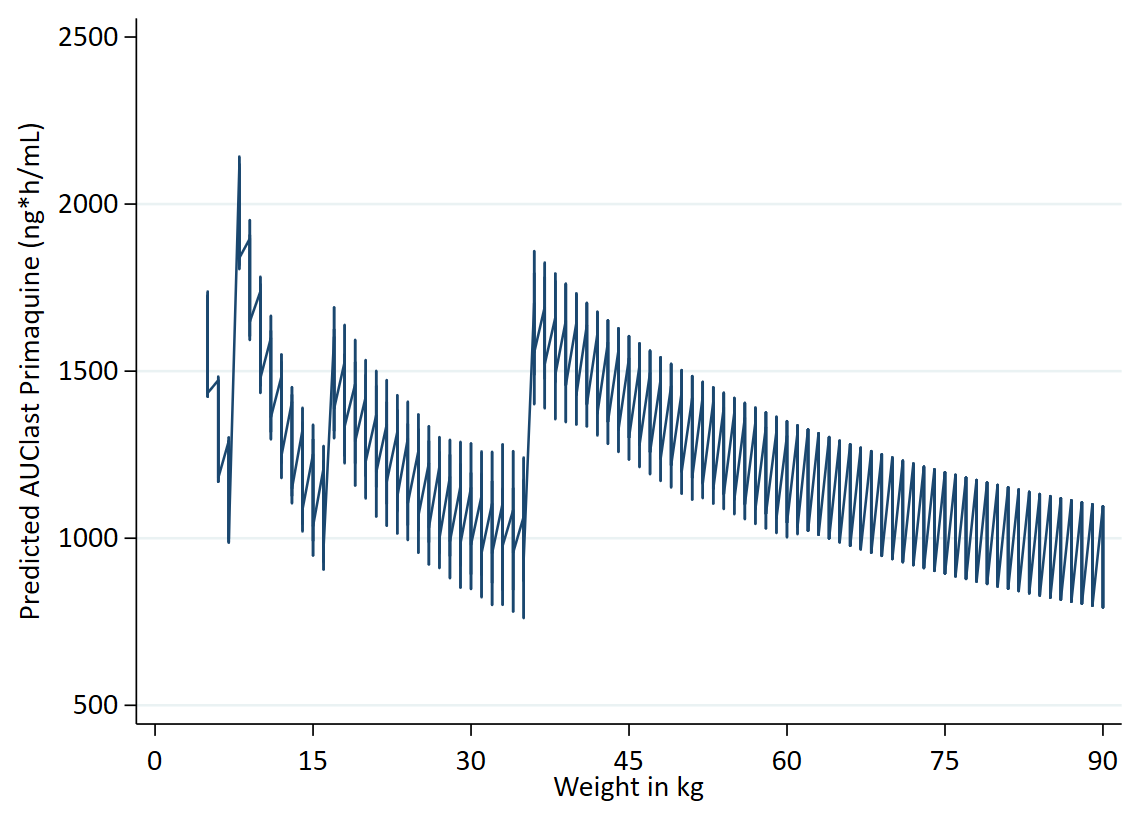** | \| **Weight** \| **Dose** \| **mg/kg** \| \| \| --- \| --- \| --- \| --- \| \| 5 to 7.9 \| 2.5 \| 0.32 \| 0.50 \| \| 8 to 10.9 \| 5 \| 0.46 \| 0.63 \| \| 11 to 16.9 \| 5 \| 0.30 \| 0.45 \| \| 17 to 24.9 \| 7.5 \| 0.30 \| 0.44 \| \| 25 to 35.9 \| 7.5 \| 0.21 \| 0.30 \| \| 36 to 59.9 \| 15 \| 0.25 \| 0.42 \| \| 60 to 79.9 \| 15 \| 0.19 \| 0.25 \| \| 80 to 90 \| 15 \| 0.17 \| 0.19 \| |
| --- | --- | --- | --- | --- | --- | --- | --- | --- | --- | --- | --- | --- | --- | --- | --- | --- | --- | --- | --- | --- | --- | --- | --- | --- | --- | --- | --- | --- | --- | --- | --- | --- | --- | --- | --- | --- | --- |

## Primaquine AUC for DHAPP – using 30 mg in band 8.

| **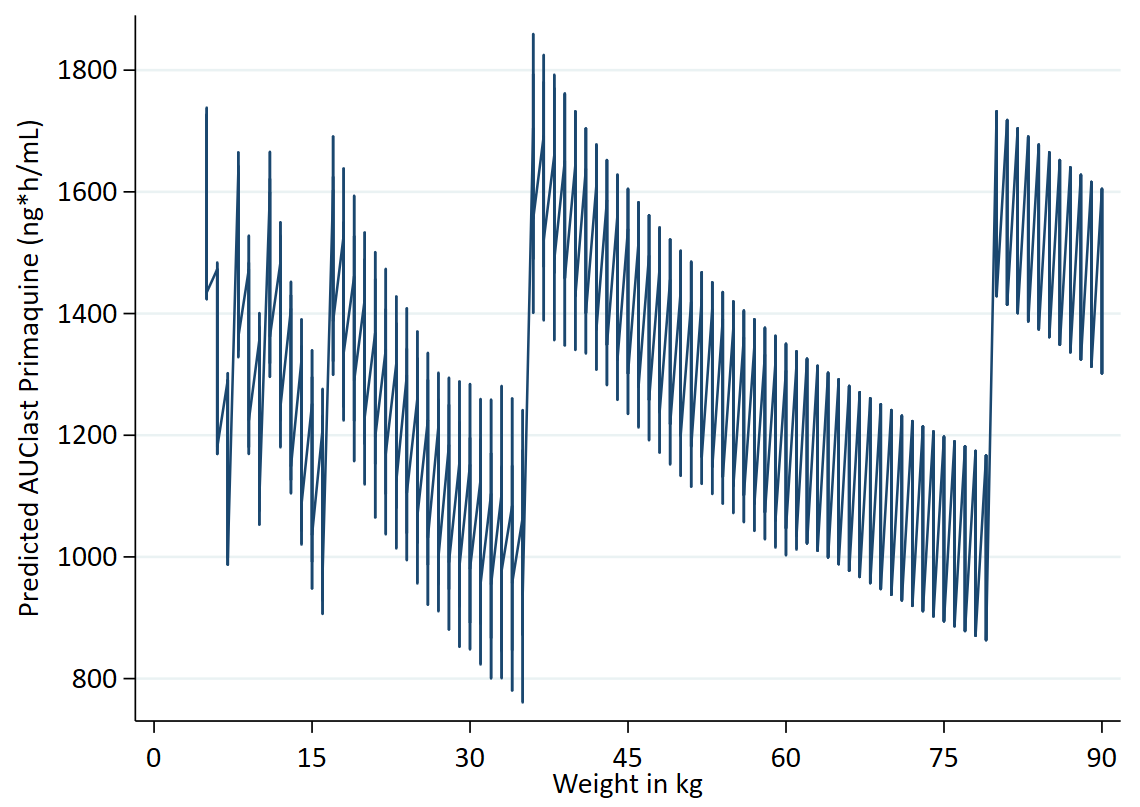** | \| **Weight** \| **Dose** \| **mg/kg** \| \| \| --- \| --- \| --- \| --- \| \| 5 to 7.9 \| 2.5 \| 0.32 \| 0.50 \| \| 8 to 10.9 \| 3.75 \| 0.34 \| 0.47 \| \| 11 to 16.9 \| 5 \| 0.30 \| 0.45 \| \| 17 to 24.9 \| 7.5 \| 0.30 \| 0.44 \| \| 25 to 35.9 \| 7.5 \| 0.21 \| 0.30 \| \| 36 to 59.9 \| 15 \| 0.25 \| 0.42 \| \| 60 to 79.9 \| 15 \| 0.19 \| 0.25 \| \| 80 to 90 \| 30 \| 0.33 \| 0.38 \| |
| --- | --- | --- | --- | --- | --- | --- | --- | --- | --- | --- | --- | --- | --- | --- | --- | --- | --- | --- | --- | --- | --- | --- | --- | --- | --- | --- | --- | --- | --- | --- | --- | --- | --- | --- | --- | --- | --- |

#

# AUC ASPYR.

## Primaquine AUC for ASPYR – full line.

| **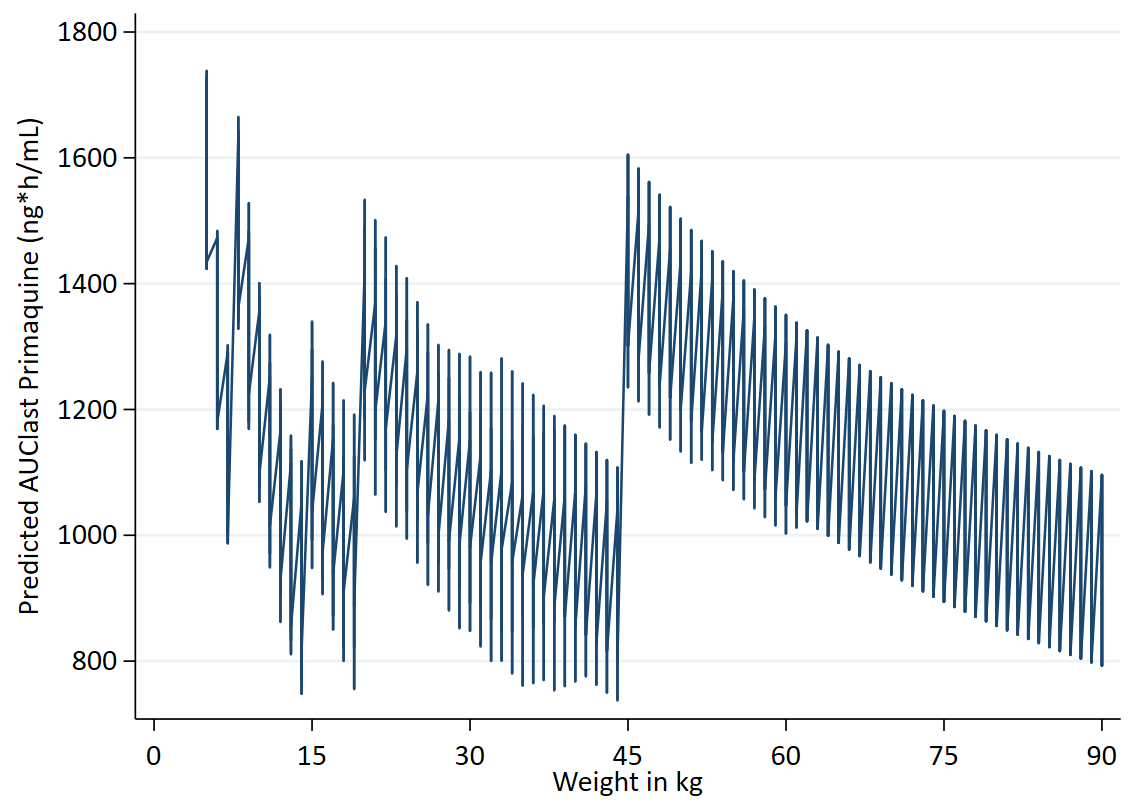** | \| **Weight** \| **Dose** \| **mg/kg** \| \| \| --- \| --- \| --- \| --- \| \| 5 to 7.9 \| 2.5 \| 0.32 \| 0.50 \| \| 8 to 14.9 \| 3.75 \| 0.25 \| 0.47 \| \| 15 to 19.9 \| 5 \| 0.25 \| 0.33 \| \| 20 to 23.9 \| 7.5 \| 0.31 \| 0.38 \| \| 24 to 44.9 \| 7.5 \| 0.17 \| 0.31 \| \| 45 to 64.9 \| 15 \| 0.23 \| 0.33 \| \| 65 to 90 \| 15 \| 0.17 \| 0.23 \| |
| --- | --- | --- | --- | --- | --- | --- | --- | --- | --- | --- | --- | --- | --- | --- | --- | --- | --- | --- | --- | --- | --- | --- | --- | --- | --- | --- | --- | --- | --- | --- | --- | --- | --- |

## Primaquine AUC for ASPYR – 5 mg replaces 3.75 mg in band 2.

| **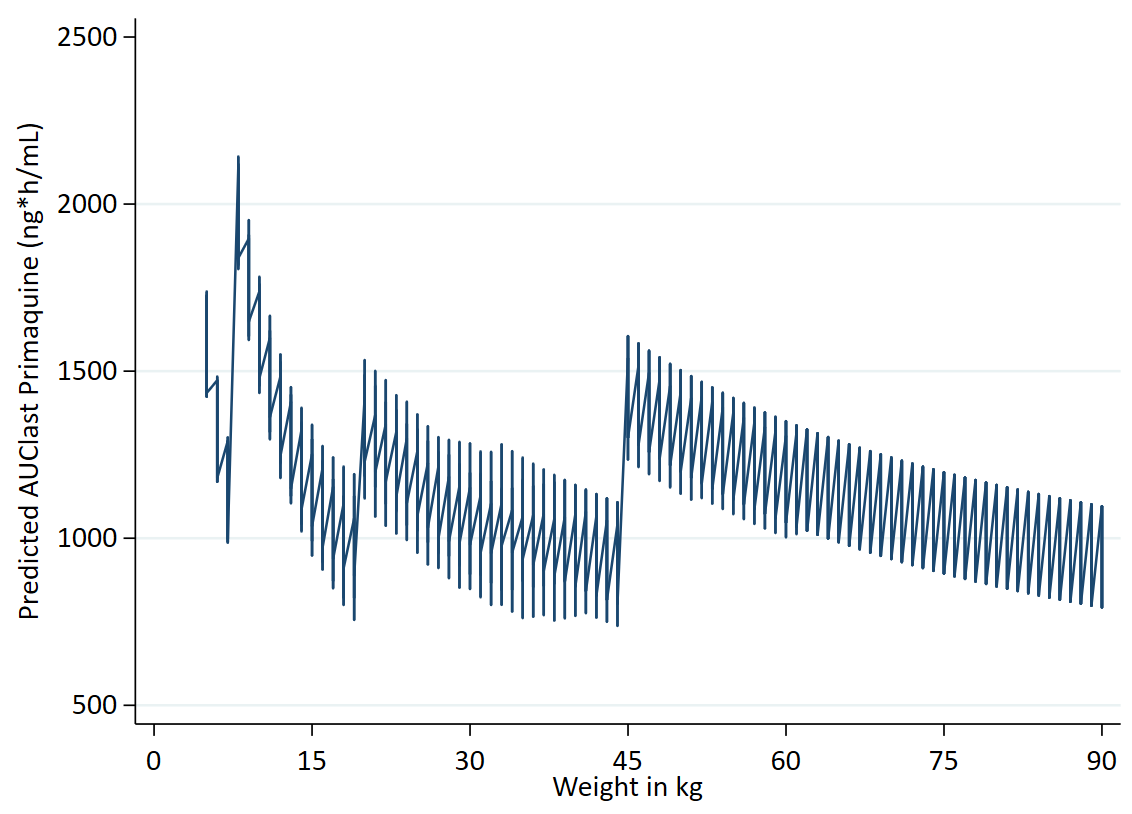** | \| **Weight** \| **Dose** \| **mg/kg** \| \| \| --- \| --- \| --- \| --- \| \| 5 to 7.9 \| 2.5 \| 0.32 \| 0.50 \| \| 8 to 14.9 \| 5 \| 0.34 \| 0.63 \| \| 15 to 19.9 \| 5 \| 0.25 \| 0.33 \| \| 20 to 23.9 \| 7.5 \| 0.31 \| 0.38 \| \| 24 to 44.9 \| 7.5 \| 0.17 \| 0.31 \| \| 45 to 64.9 \| 15 \| 0.23 \| 0.33 \| \| 65 to 90 \| 15 \| 0.17 \| 0.23 \| |
| --- | --- | --- | --- | --- | --- | --- | --- | --- | --- | --- | --- | --- | --- | --- | --- | --- | --- | --- | --- | --- | --- | --- | --- | --- | --- | --- | --- | --- | --- | --- | --- | --- | --- |

## Primaquine AUC for ASPYR – 7.5 mg replaces 5 mg in band 3.

| **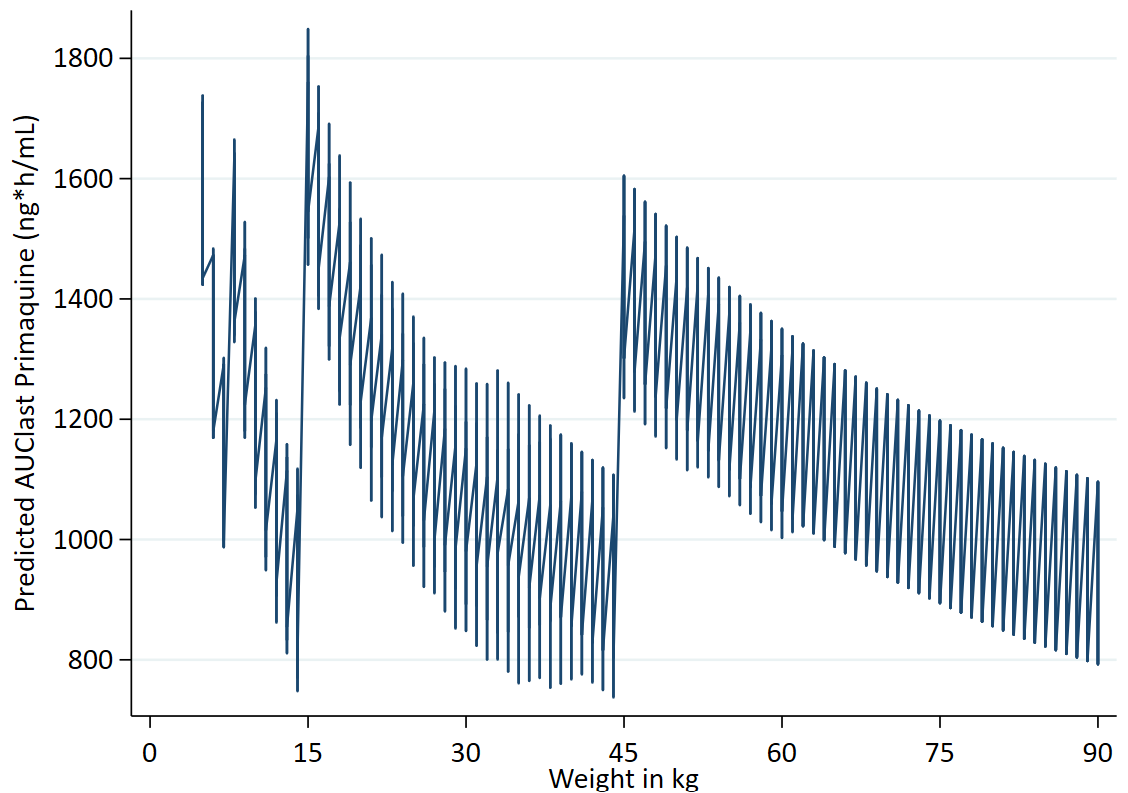** | \| **Weight** \| **Dose** \| **mg/kg** \| \| \| --- \| --- \| --- \| --- \| \| 5 to 7.9 \| 2.5 \| 0.32 \| 0.50 \| \| 8 to 14.9 \| 3.75 \| 0.25 \| 0.47 \| \| 15 to 19.9 \| 7.5 \| 0.38 \| 0.5 \| \| 20 to 23.9 \| 7.5 \| 0.31 \| 0.38 \| \| 24 to 44.9 \| 7.5 \| 0.17 \| 0.31 \| \| 45 to 64.9 \| 15 \| 0.23 \| 0.33 \| \| 65 to 90 \| 15 \| 0.17 \| 0.23 \| |
| --- | --- | --- | --- | --- | --- | --- | --- | --- | --- | --- | --- | --- | --- | --- | --- | --- | --- | --- | --- | --- | --- | --- | --- | --- | --- | --- | --- | --- | --- | --- | --- | --- | --- |

## Primaquine AUC for ASPYR – using 11.25 mg in band 5.

| **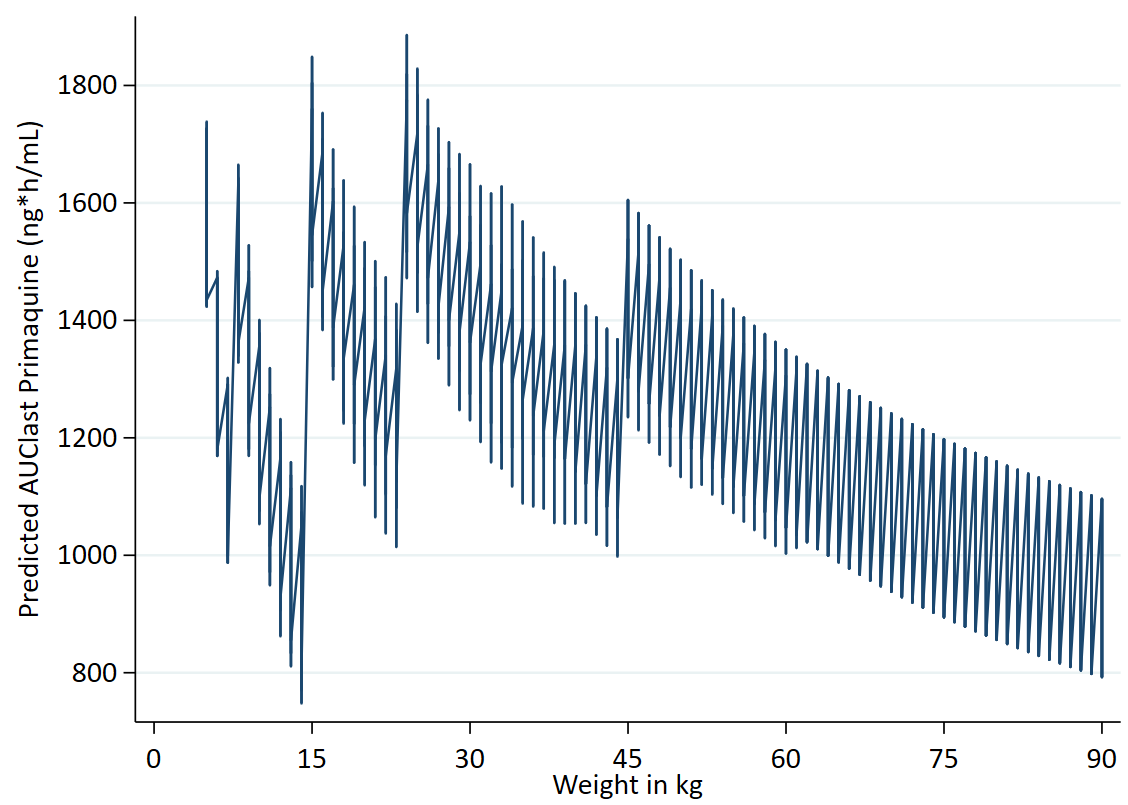** | \| **Weight** \| **Dose** \| **mg/kg** \| \| \| --- \| --- \| --- \| --- \| \| 5 to 7.9 \| 2.5 \| 0.32 \| 0.50 \| \| 8 to 14.9 \| 3.75 \| 0.25 \| 0.47 \| \| 15 to 19.9 \| 7.5 \| 0.38 \| 0.5 \| \| 20 to 23.9 \| 7.5 \| 0.31 \| 0.38 \| \| 24 to 44.9 \| 11.25 \| 0.25 \| 0.47 \| \| 45 to 64.9 \| 15 \| 0.23 \| 0.33 \| \| 65 to 90 \| 15 \| 0.17 \| 0.23 \| |
| --- | --- | --- | --- | --- | --- | --- | --- | --- | --- | --- | --- | --- | --- | --- | --- | --- | --- | --- | --- | --- | --- | --- | --- | --- | --- | --- | --- | --- | --- | --- | --- | --- | --- |

# AUC ALAQ triple ACT.

## Primaquine AUC for ALAQ – using 3.75 mg.

| **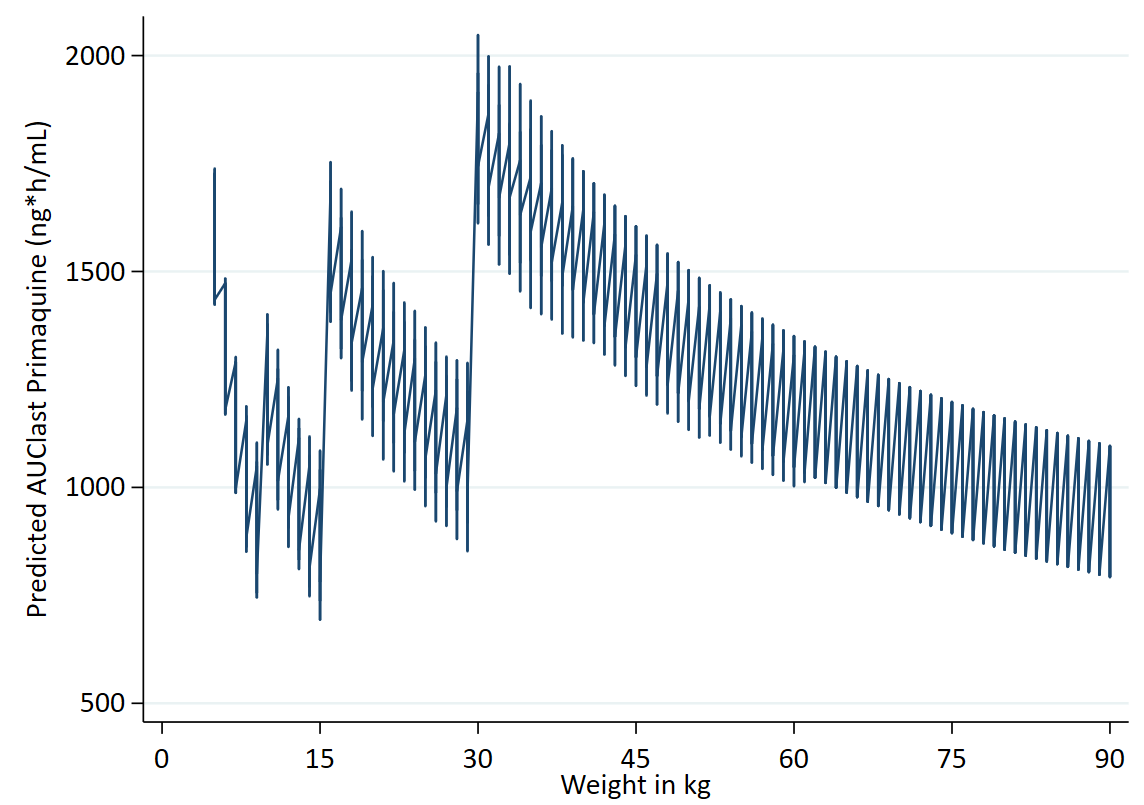** | \| **Weight** \| **Dose** \| **mg/kg** \| \| \| --- \| --- \| --- \| --- \| \| 5 to 9.9 \| 2.5 \| 0.25 \| 0.50 \| \| 10 to 15.9 \| 3.75 \| 0.24 \| 0.38 \| \| 16 to 29.9 \| 7.5 \| 0.25 \| 0.47 \| \| 30 to 54.9 \| 15 \| 0.27 \| 0.50 \| \| 55 to 90 \| 15 \| 0.17 \| 0.27 \| |
| --- | --- | --- | --- | --- | --- | --- | --- | --- | --- | --- | --- | --- | --- | --- | --- | --- | --- | --- | --- | --- | --- | --- | --- | --- | --- |

## Primaquine AUC for ALAQ – 5 mg replaces 3.75 mg in band 2.

| **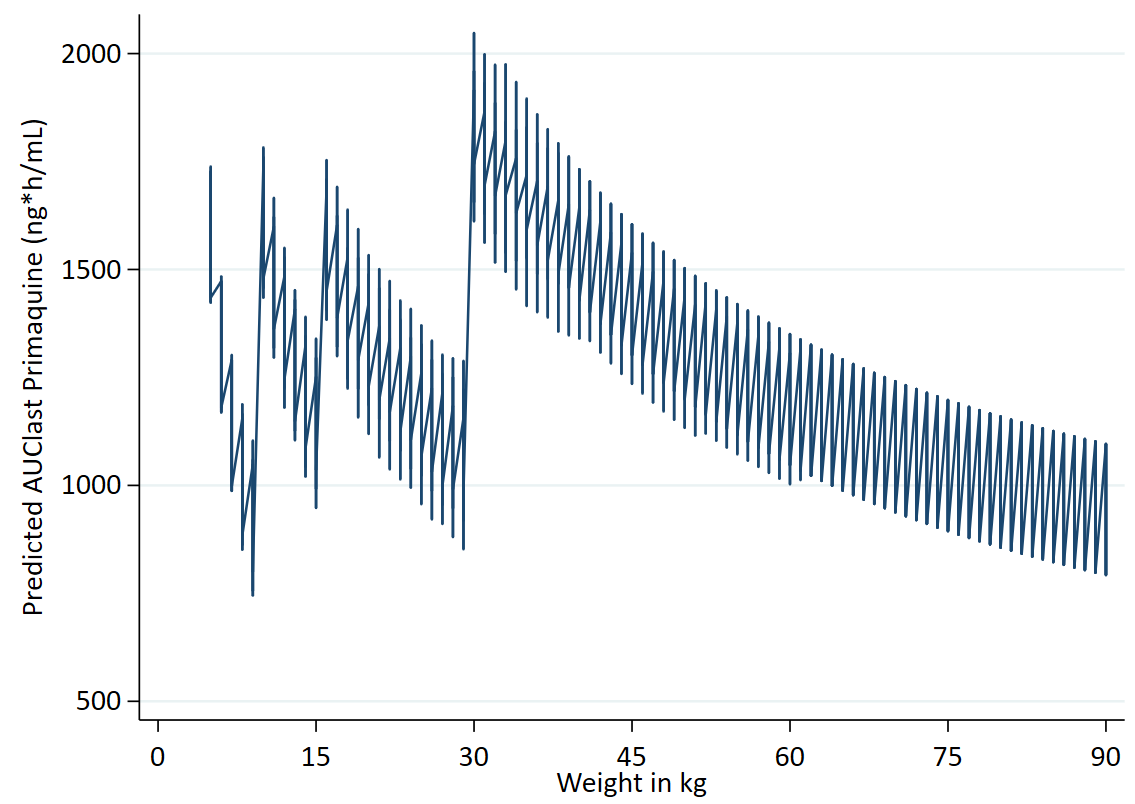** | \| **Weight** \| **Dose** \| **mg/kg** \| \| \| --- \| --- \| --- \| --- \| \| 5 to 9.9 \| 2.5 \| 0.25 \| 0.50 \| \| 10 to 15.9 \| 5 \| 0.31 \| 0.5 \| \| 16 to 29.9 \| 7.5 \| 0.25 \| 0.47 \| \| 30 to 54.9 \| 15 \| 0.27 \| 0.50 \| \| 55 to 90 \| 15 \| 0.17 \| 0.27 \| |
| --- | --- | --- | --- | --- | --- | --- | --- | --- | --- | --- | --- | --- | --- | --- | --- | --- | --- | --- | --- | --- | --- | --- | --- | --- | --- |

## Primaquine AUC for ALAQ – using 11.25 mg in band 4.

| **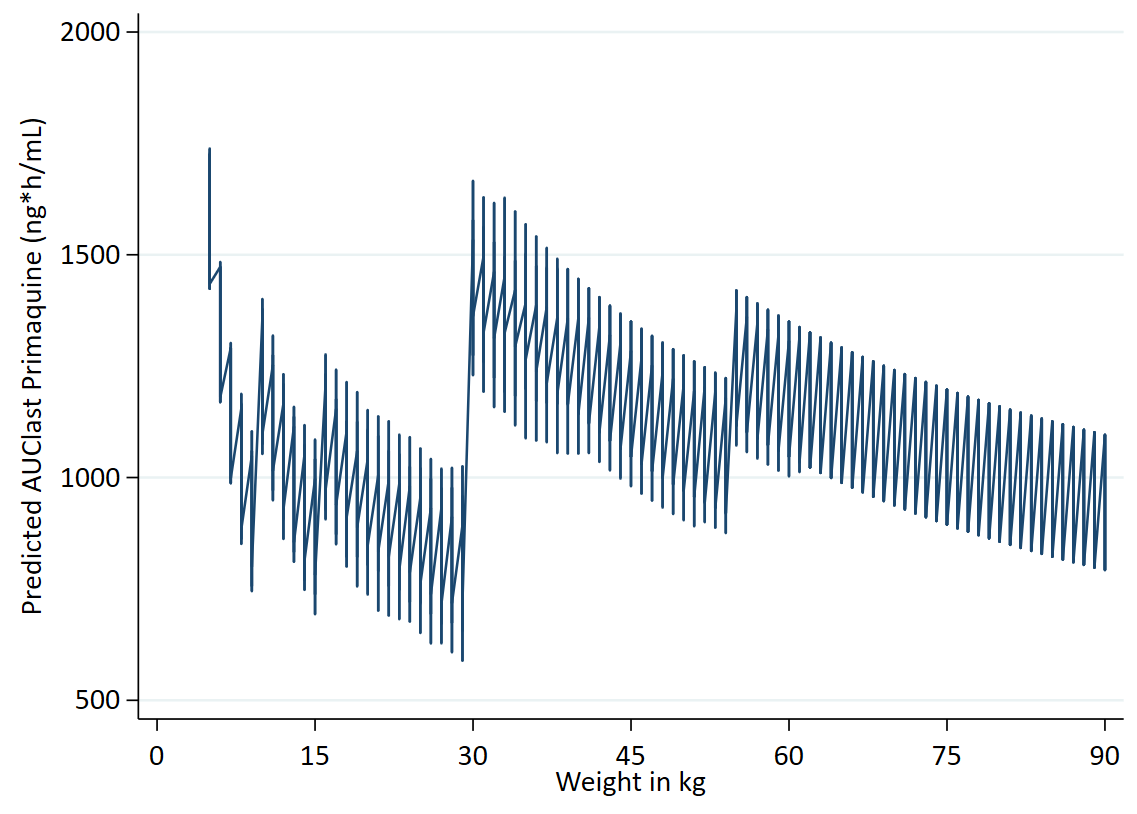** | \| **Weight** \| **Dose** \| **mg/kg** \| \| \| --- \| --- \| --- \| --- \| \| 5 to 9.9 \| 2.5 \| 0.25 \| 0.50 \| \| 10 to 15.9 \| 3.75 \| 0.24 \| 0.38 \| \| 16 to 29.9 \| 7.5 \| 0.17 \| 0.31 \| \| 30 to 54.9 \| 11.25 \| 0.20 \| 0.38 \| \| 55 to 90 \| 15 \| 0.17 \| 0.27 \| |
| --- | --- | --- | --- | --- | --- | --- | --- | --- | --- | --- | --- | --- | --- | --- | --- | --- | --- | --- | --- | --- | --- | --- | --- | --- | --- |

## Primaquine AUC for ALAQ – using 22.5 mg in band 5.

| **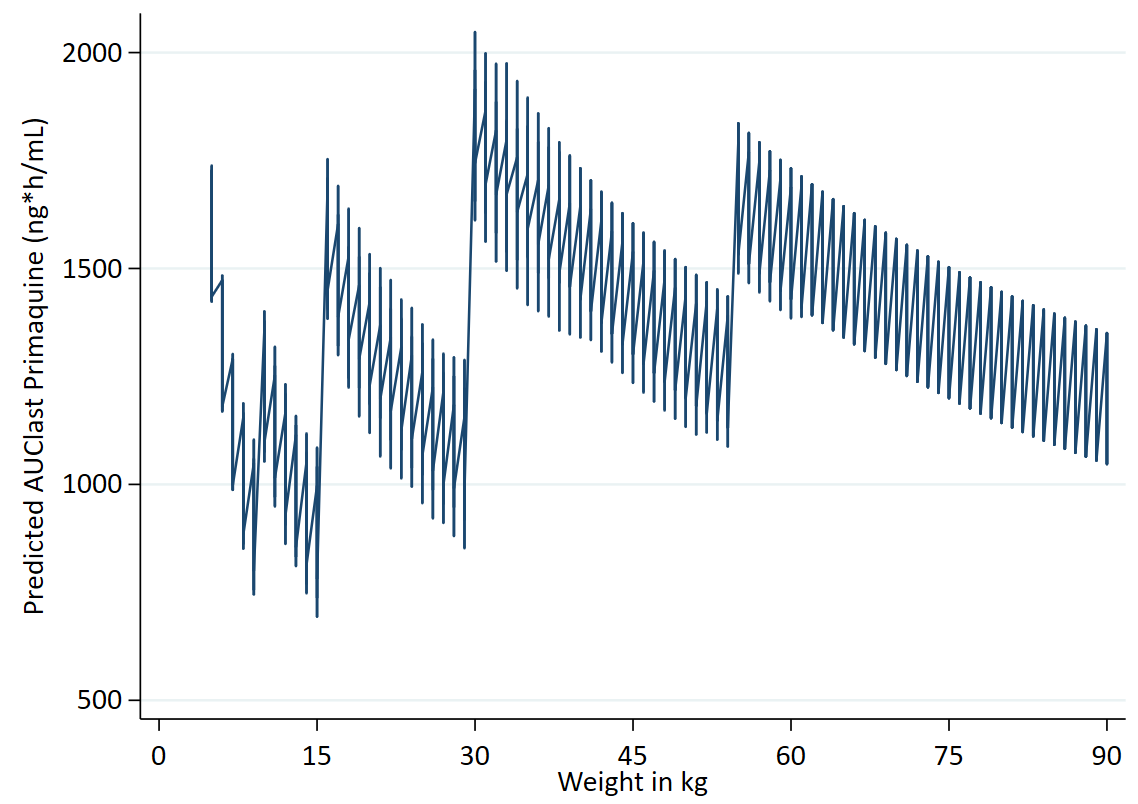** | \| **Weight** \| **Dose** \| **mg/kg** \| \| \| --- \| --- \| --- \| --- \| \| 5 to 9.9 \| 2.5 \| 0.25 \| 0.50 \| \| 10 to 15.9 \| 3.75 \| 0.24 \| 0.38 \| \| 16 to 29.9 \| 7.5 \| 0.25 \| 0.47 \| \| 30 to 54.9 \| 15 \| 0.27 \| 0.50 \| \| 55 to 90 \| 22.5 \| 0.25 \| 0.41 \| |
| --- | --- | --- | --- | --- | --- | --- | --- | --- | --- | --- | --- | --- | --- | --- | --- | --- | --- | --- | --- | --- | --- | --- | --- | --- | --- |

# AUC ASAQ.

## Primaquine AUC for ASAQ – using 3.75 mg.

| **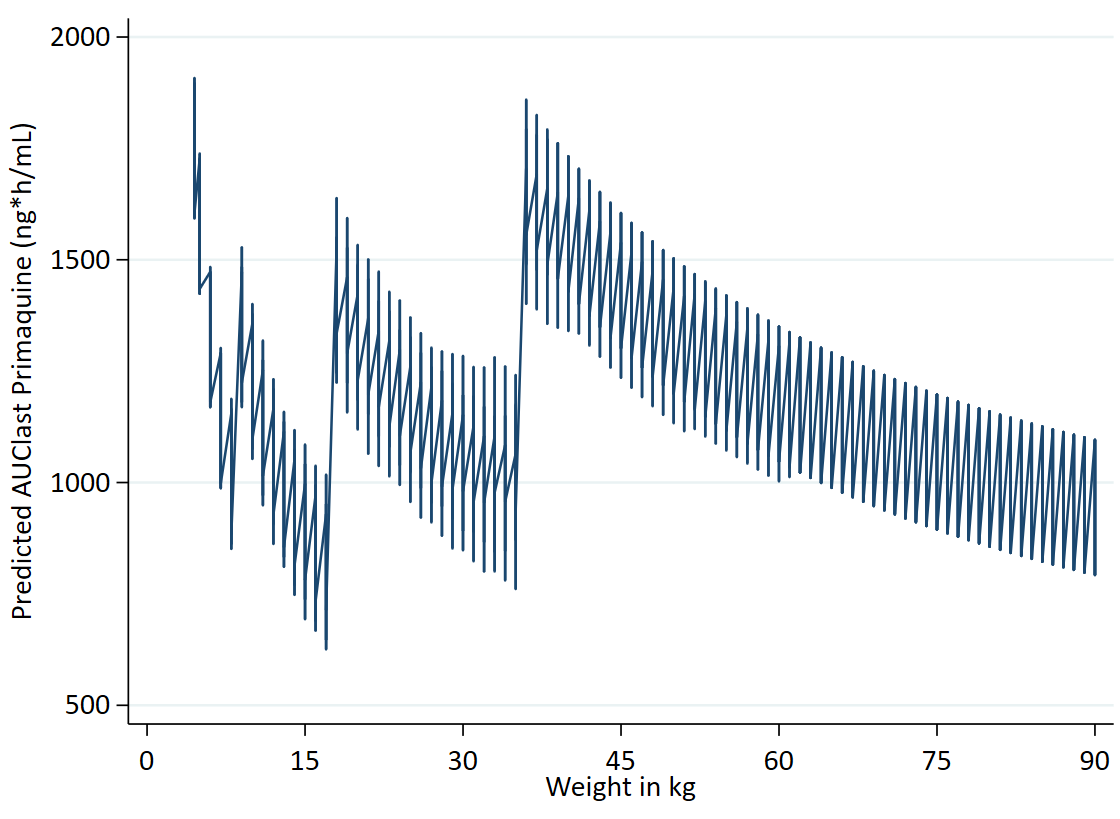** | \| **Weight** \| **Dose** \| **mg/kg** \| \| \| --- \| --- \| --- \| --- \| \| 4.5 to 8.9 \| 2.5 \| 0.28 \| 0.56 \| \| 9 to 17.9 \| 3.75 \| 0.21 \| 0.42 \| \| 18 to 35.9 \| 7.5 \| 0.21 \| 0.42 \| \| 36 to 90 \| 15 \| 0.17 \| 0.42 \| |
| --- | --- | --- | --- | --- | --- | --- | --- | --- | --- | --- | --- | --- | --- | --- | --- | --- | --- | --- | --- | --- | --- |

## Primaquine AUC for ASAQ – 5 mg replaces 3.75 mg in band 2.

| **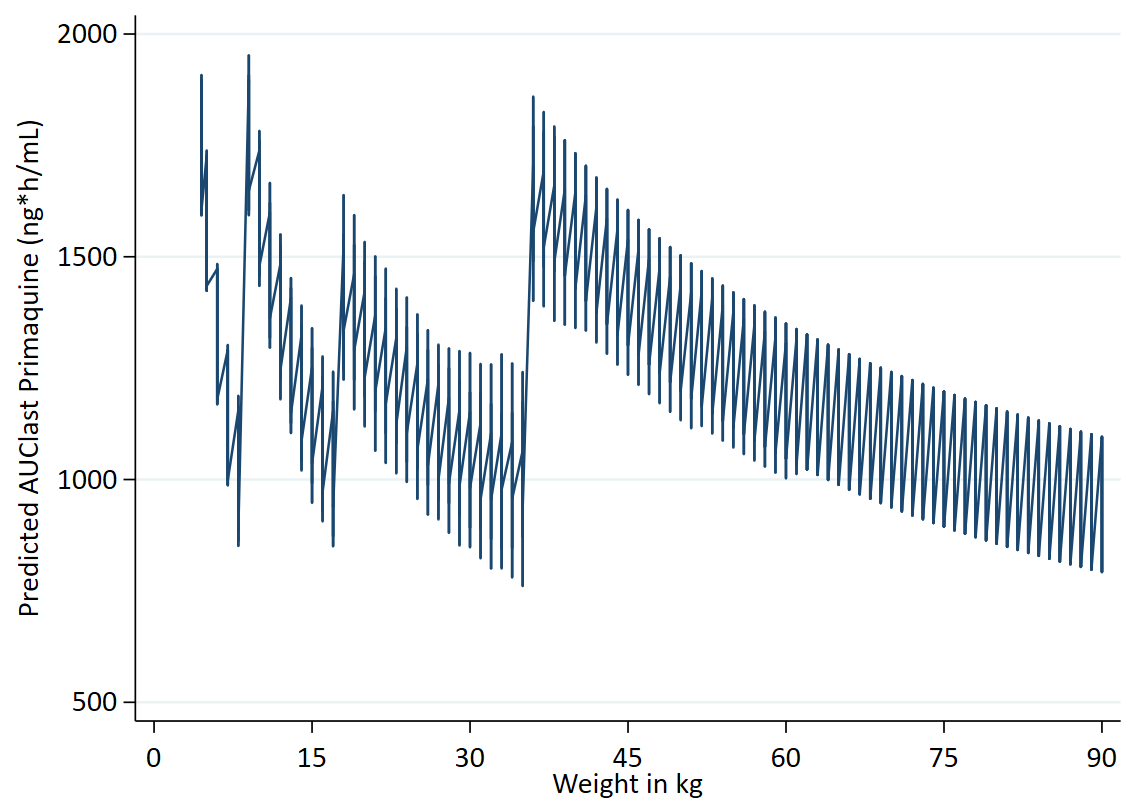** | \| **Weight** \| **Dose** \| **mg/kg** \| \| \| --- \| --- \| --- \| --- \| \| 4.5 to 8.9 \| 2.5 \| 0.28 \| 0.56 \| \| 9 to 17.9 \| 5 \| 0.28 \| 0.56 \| \| 18 to 35.9 \| 7.5 \| 0.21 \| 0.42 \| \| 36 to 90 \| 15 \| 0.17 \| 0.42 \| |
| --- | --- | --- | --- | --- | --- | --- | --- | --- | --- | --- | --- | --- | --- | --- | --- | --- | --- | --- | --- | --- | --- |

# AUC ASMQ

## Primaquine AUC for ASMQ – using 3.75 mg.

| 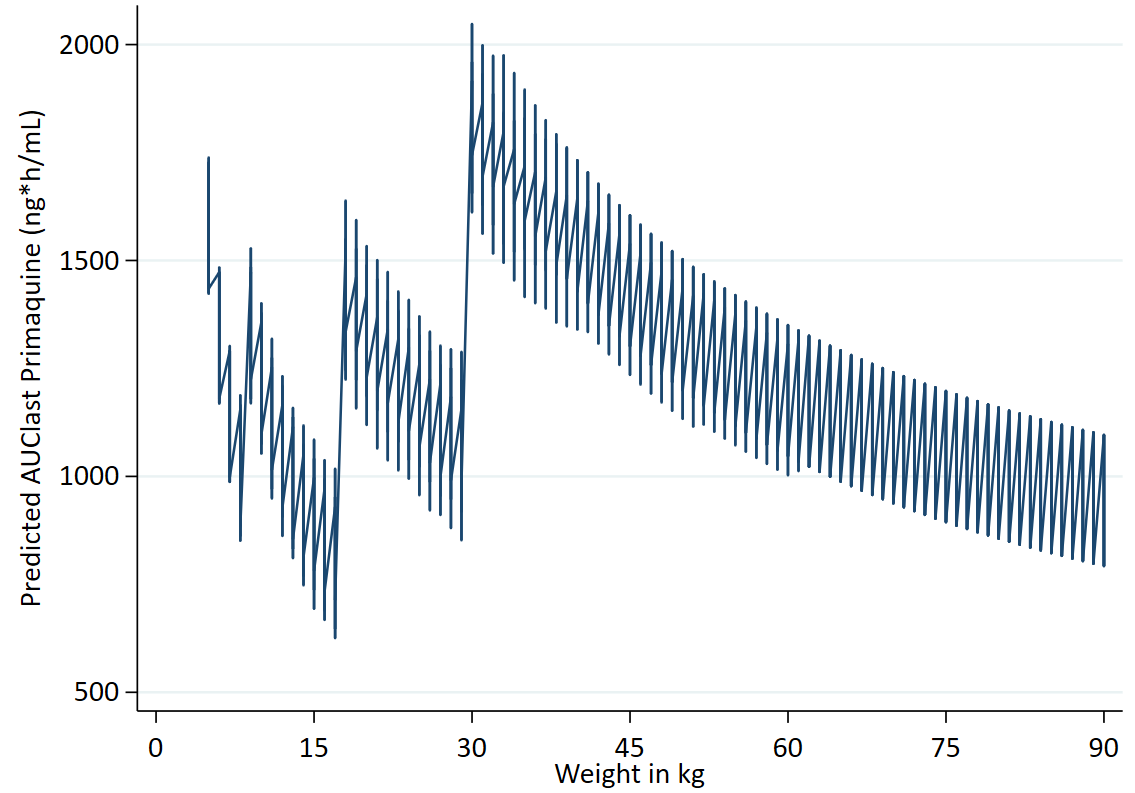 | \| **Weight** \| **Dose** \| **mg/kg** \| \| \| --- \| --- \| --- \| --- \| \| 5 to 8.9 \| 2.5 \| 0.28 \| 0.50 \| \| 9 to 17.9 \| 3.75 \| 0.21 \| 0.42 \| \| 18 to 29.9 \| 7.5 \| 0.25 \| 0.42 \| \| 30 to 90 \| 15 \| 0.17 \| 0.50 \| |
| --- | --- | --- | --- | --- | --- | --- | --- | --- | --- | --- | --- | --- | --- | --- | --- | --- | --- | --- | --- | --- | --- |

## Primaquine AUC for ASMQ – 5 mg replaces 3.75 mg in band 2.

| 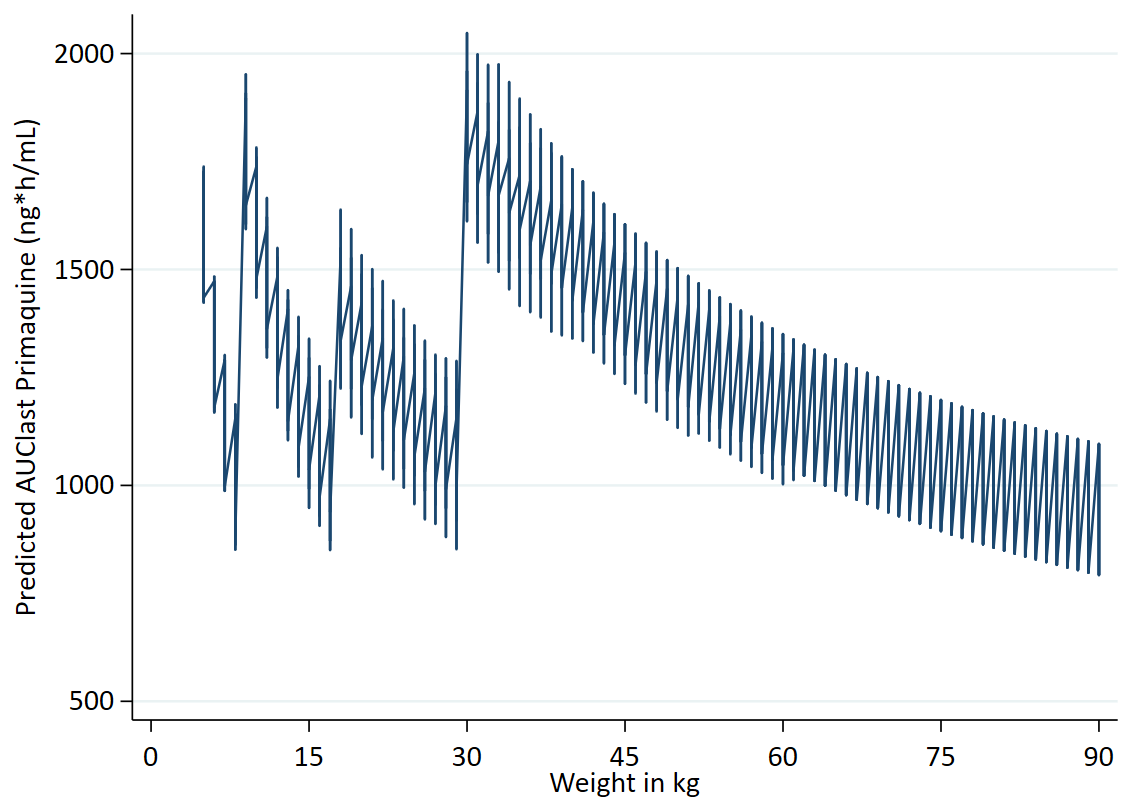 | \| **Weight** \| **Dose** \| **mg/kg** \| \| \| --- \| --- \| --- \| --- \| \| 5 to 8.9 \| 2.5 \| 0.28 \| 0.50 \| \| 9 to 17.9 \| 5 \| 0.28 \| 0.56 \| \| 18 to 29.9 \| 7.5 \| 0.25 \| 0.42 \| \| 30 to 90 \| 15 \| 0.17 \| 0.50 \| |
| --- | --- | --- | --- | --- | --- | --- | --- | --- | --- | --- | --- | --- | --- | --- | --- | --- | --- | --- | --- | --- | --- |

# AUC AL.

## Primaquine AUCs for two SLDPQ options: 5 or 7.5 mg in band 2.

| 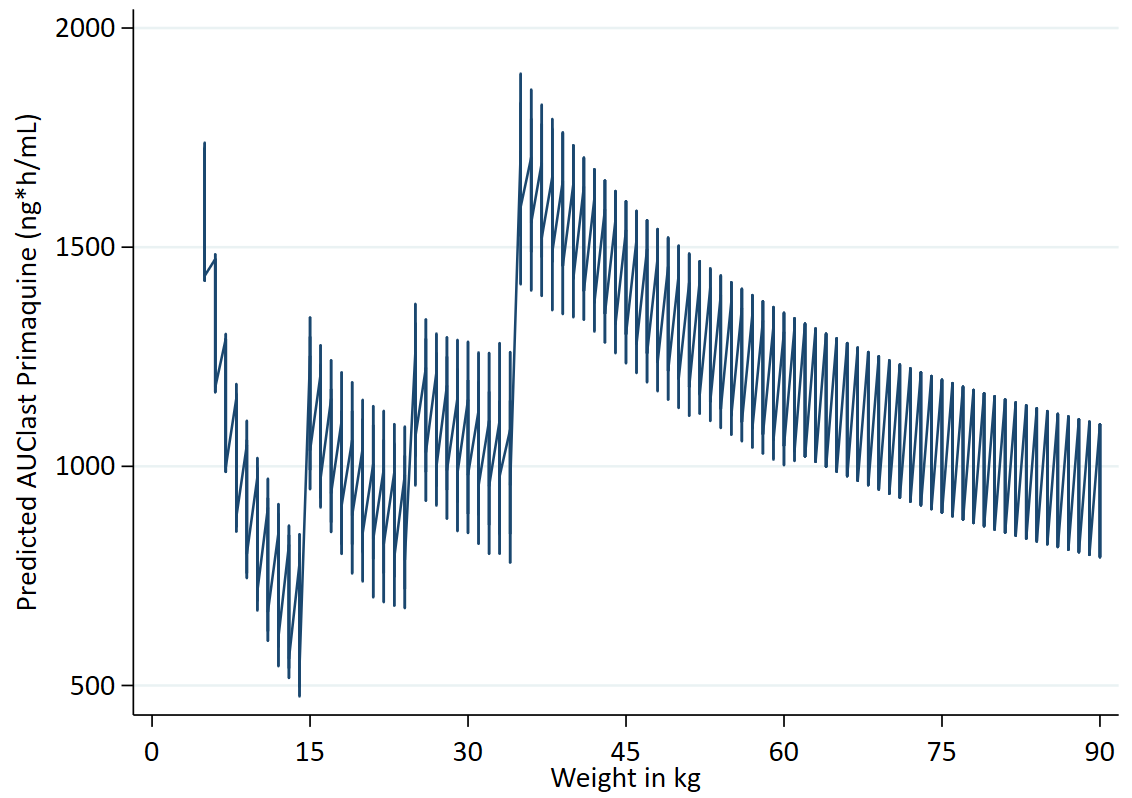 | \| **Weight** \| **Dose** \| **mg/kg** \| \| \| --- \| --- \| --- \| --- \| \| 5 to 14.9 \| 2.5 \| 0.17 \| 0.50 \| \| 15 to 24.9 \| 5 \| 0.20 \| 0.33 \| \| 25 to 34.9 \| 7.5 \| 0.21 \| 0.30 \| \| 35 to 90 \| 15 \| 0.17 \| 0.43 \| |
| --- | --- | --- | --- | --- | --- | --- | --- | --- | --- | --- | --- | --- | --- | --- | --- | --- | --- | --- | --- | --- | --- |
| 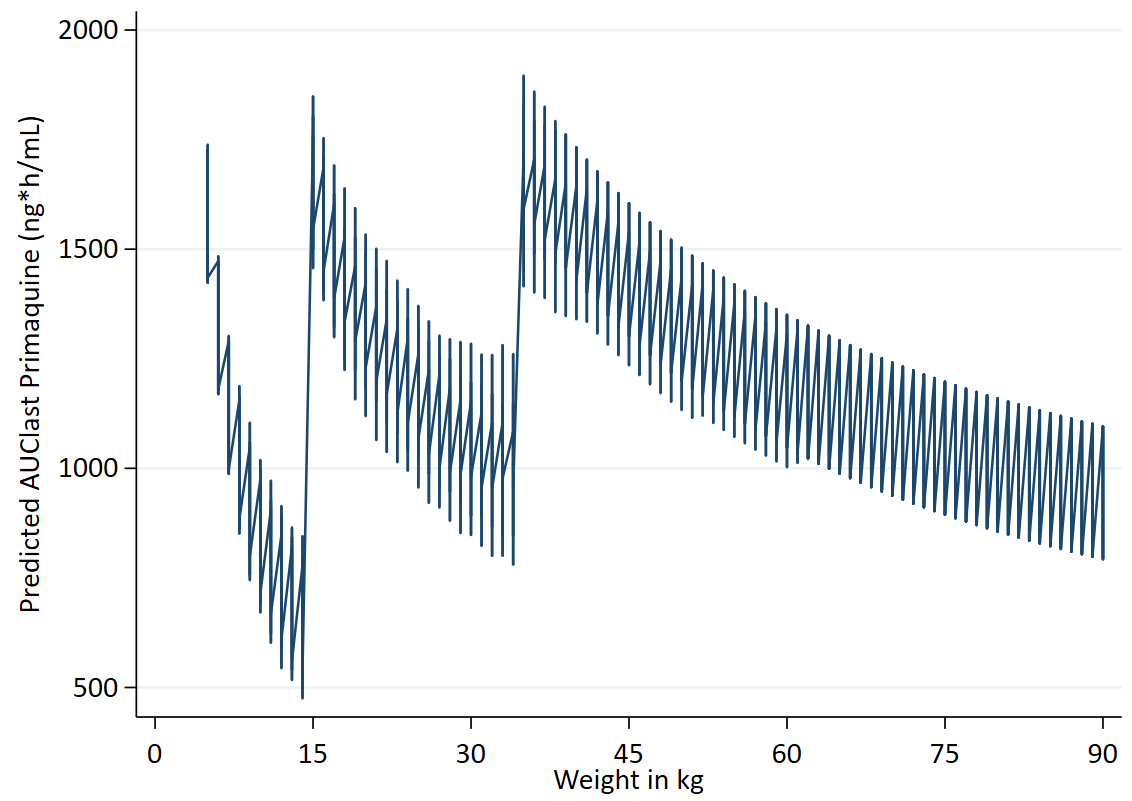 | \| **Weight** \| **Dose** \| **mg/kg** \| \| \| --- \| --- \| --- \| --- \| \| 5 to 14.9 \| 2.5 \| 0.17 \| 0.50 \| \| 15 to 24.9 \| 7.5 \| 0.30 \| 0.50 \| \| 25 to 34.9 \| 7.5 \| 0.21 \| 0.30 \| \| 35 to 90 \| 15 \| 0.17 \| 0.43 \| |

# AUC for SLDPQ matched to a *P. vivax* radical cure regimen.

## Primaquine AUC vivax-matched – using 3.75 mg.

| 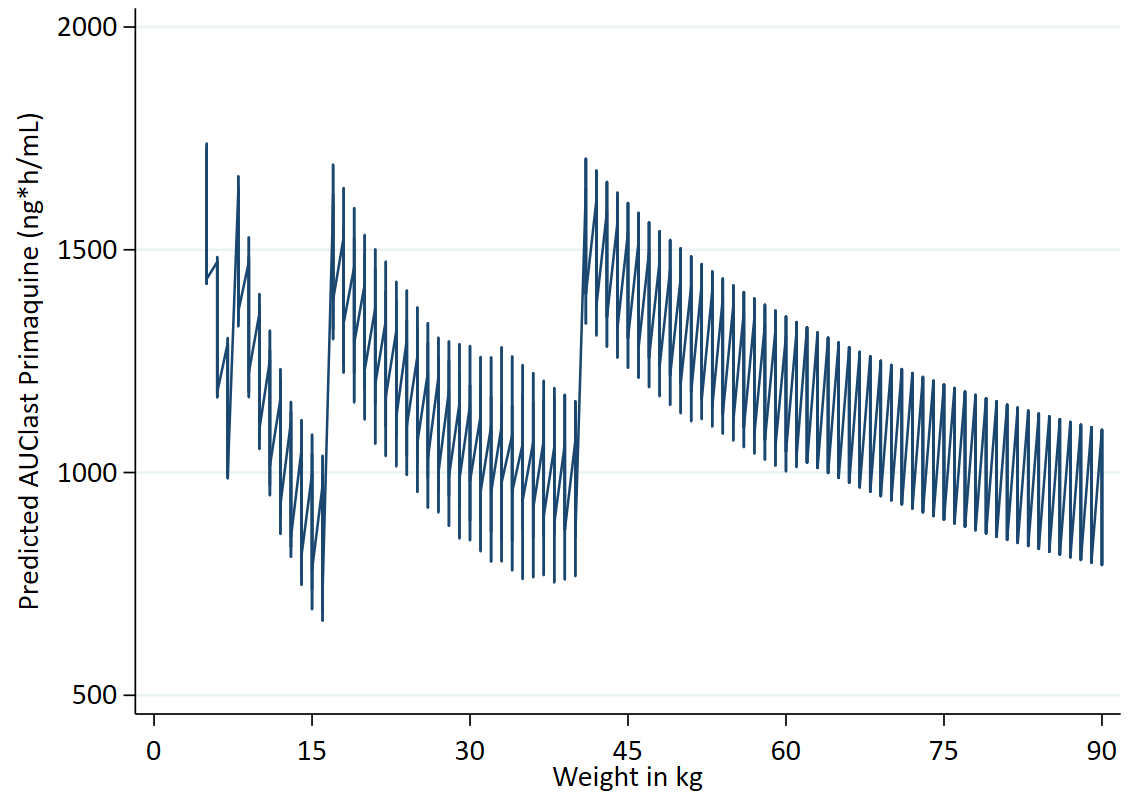 | \| **Weight** \| **Dose** \| **mg/kg** \| \| \| --- \| --- \| --- \| --- \| \| 5 to 7.9 \| 2.5 \| 0.32 \| 0.50 \| \| 8 to 16.9 \| 3.75 \| 0.22 \| 0.47 \| \| 17 to 40.9 \| 7.5 \| 0.18 \| 0.44 \| \| 41 to 80.9 \| 15 \| 0.19 \| 0.37 \| \| 81 to 90 \| 15 \| 0.17 \| 0.19 \| |
| --- | --- | --- | --- | --- | --- | --- | --- | --- | --- | --- | --- | --- | --- | --- | --- | --- | --- | --- | --- | --- | --- | --- | --- | --- | --- |

## Primaquine AUC vivax-matched – 5 mg replaces 3.75 mg in band 2.

| 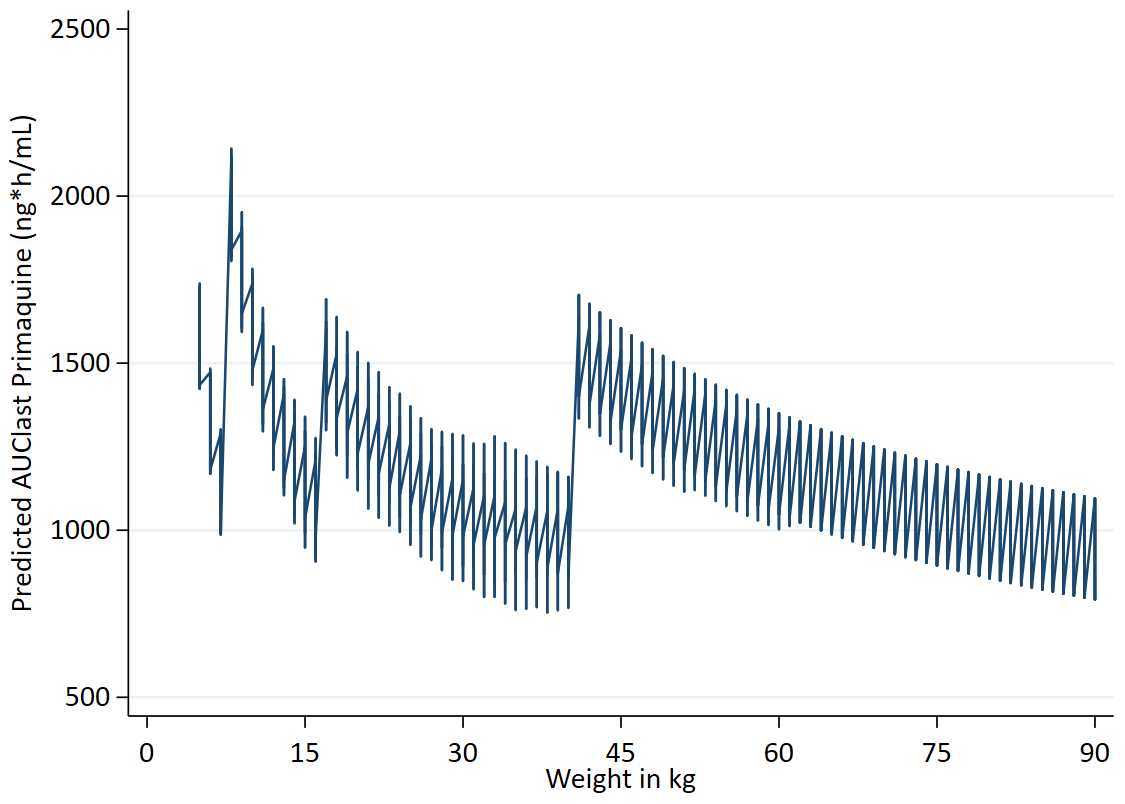 | \| **Weight** \| **Dose** \| **mg/kg** \| \| \| --- \| --- \| --- \| --- \| \| 5 to 7.9 \| 2.5 \| 0.32 \| 0.50 \| \| 8 to 16.9 \| 5 \| 0.3 \| 0.63 \| \| 17 to 40.9 \| 7.5 \| 0.18 \| 0.44 \| \| 41 to 80.9 \| 15 \| 0.19 \| 0.37 \| \| 81 to 90 \| 15 \| 0.17 \| 0.19 \| |
| --- | --- | --- | --- | --- | --- | --- | --- | --- | --- | --- | --- | --- | --- | --- | --- | --- | --- | --- | --- | --- | --- | --- | --- | --- | --- |

## Primaquine AUCs vivax-matched – using 22.5 & 30 mg in the last weight band.

| **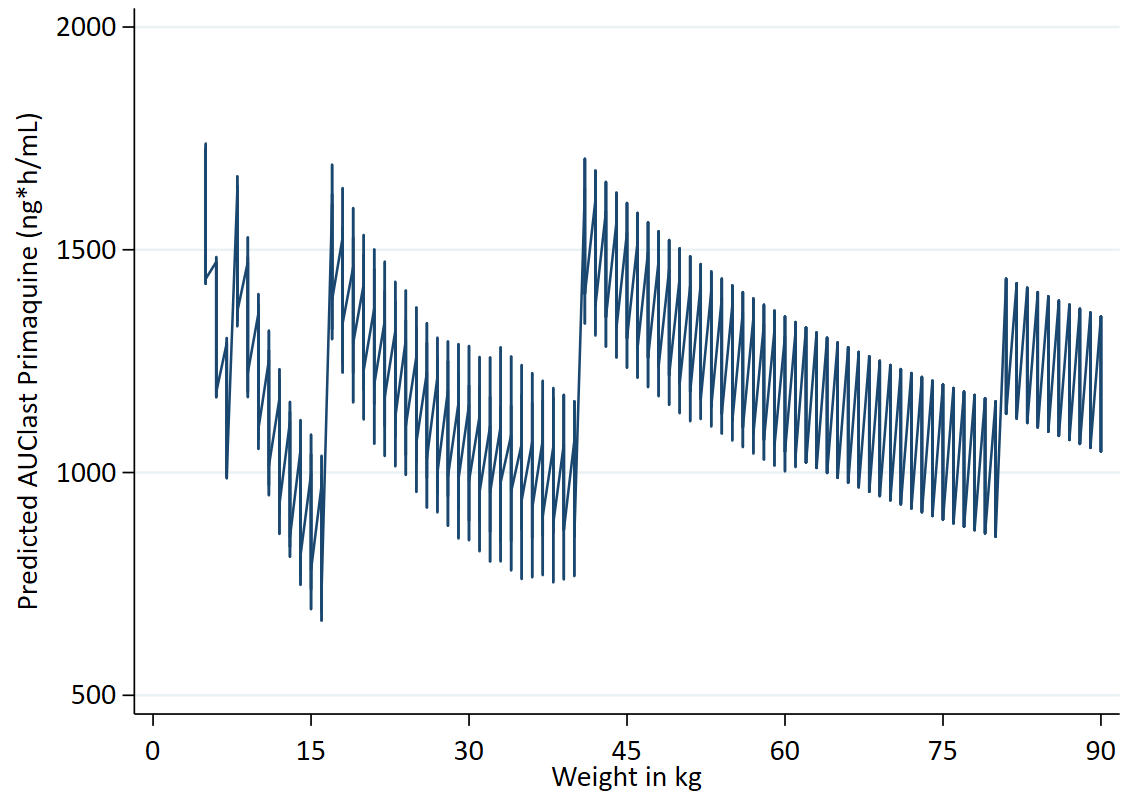** | \| **Weight** \| **Dose** \| **mg/kg** \| \| \| --- \| --- \| --- \| --- \| \| 5 to 7.9 \| 2.5 \| 0.32 \| 0.50 \| \| 8 to 16.9 \| 3.75 \| 0.22 \| 0.47 \| \| 17 to 40.9 \| 7.5 \| 0.18 \| 0.44 \| \| 41 to 80.9 \| 15 \| 0.19 \| 0.37 \| \| 81 to 90 \| 22.5 \| 0.25 \| 0.27 \| |
| --- | --- | --- | --- | --- | --- | --- | --- | --- | --- | --- | --- | --- | --- | --- | --- | --- | --- | --- | --- | --- | --- | --- | --- | --- | --- |
| 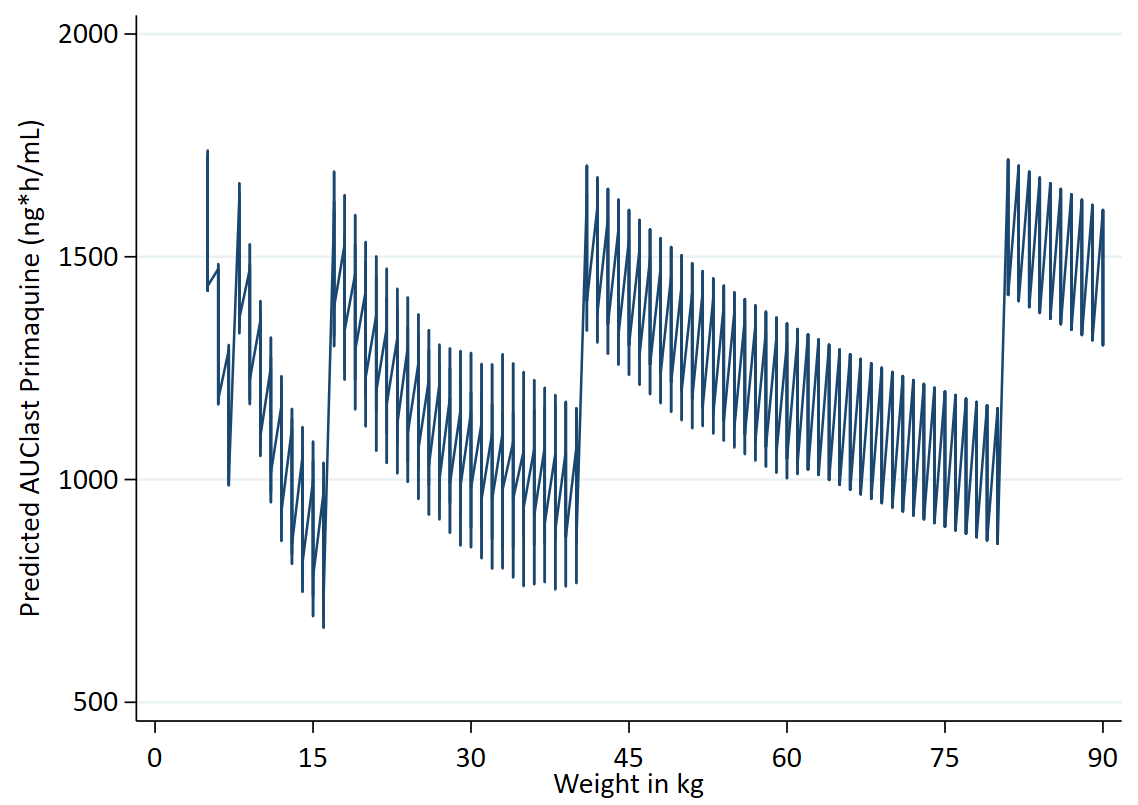 | \| **Weight** \| **Dose** \| **mg/kg** \| \| \| --- \| --- \| --- \| --- \| \| 5 to 7.9 \| 2.50 \| 0.32 \| 0.50 \| \| 8 to 16.9 \| 3.75 \| 0.22 \| 0.47 \| \| 17 to 40.9 \| 7.5 \| 0.18 \| 0.44 \| \| 41 to 80.9 \| 15 \| 0.19 \| 0.37 \| \| 81 to 90 \| 30 \| 0.33 \| 0.37 \| |

# Fig S8. Mg/kg doses of SLDPQ regimens vs. an allometric line.

# Stand-alone DPP-IMPRIMA regimen – full line.

#

|  |
| --- |
| \| **Weight** \| **Dose** \| **mg/kg** \| \| \| --- \| --- \| --- \| --- \| \| 5 to 7.9 \| 2.50 \| 0.32 \| 0.50 \| \| 8 to 11.9 \| 3.75 \| 0.32 \| 0.47 \| \| 12 to 17.9 \| 5 \| 0.28 \| 0.42 \| \| 18 to 35.9 \| 7.5 \| 0.21 \| 0.42 \| \| 36 to 69.9 \| 15 \| 0.21 \| 0.42 \| \| 70 to 90 \| 22.5 \| 0.25 \| 0.32 \| |

# Stand-alone DPP-IMPRIMA regimen mg/kg – 5 mg replaces 3.75 mg.

|  |
| --- |
| \| **Weight** \| **Dose** \| **mg/kg** \| \| \| --- \| --- \| --- \| --- \| \| 5 to 7.9 \| 2.5 \| 0.32 \| 0.50 \| \| 8 to 11.9 \| 5 \| 0.45 \| 0.63 \| \| 12 to 17.9 \| 5 \| 0.28 \| 0.42 \| \| 18 to 35.9 \| 7.5 \| 0.21 \| 0.42 \| \| 36 to 69.9 \| 15 \| 0.21 \| 0.42 \| \| 70 to 90 \| 22.5 \| 0.25 \| 0.32 \| |

# DHAPP – full line.

|  |
| --- |
| \| **Weight** \| **Dose** \| **mg/kg** \| \| \| --- \| --- \| --- \| --- \| \| 5 to 7.9 \| 2.5 \| 0.32 \| 0.50 \| \| 8 to 10.9 \| 3.75 \| 0.34 \| 0.47 \| \| 11 to 16.9 \| 5 \| 0.30 \| 0.45 \| \| 17 to 24.9 \| 7.5 \| 0.30 \| 0.44 \| \| 25 to 35.9 \| 7.5 \| 0.21 \| 0.30 \| \| 36 to 59.9 \| 15 \| 0.25 \| 0.42 \| \| 60 to 79.9 \| 15 \| 0.19 \| 0.25 \| \| 80 to 90 \| 15 \| 0.17 \| 0.19 \| |

# DHAPP – 5 mg replaces 3.75 mg.

|  |
| --- |
| \| **Weight** \| **Dose** \| **mg/kg** \| \| \| --- \| --- \| --- \| --- \| \| 5 to 7.9 \| 2.5 \| 0.32 \| 0.50 \| \| 8 to 10.9 \| 5 \| 0.46 \| 0.63 \| \| 11 to 16.9 \| 5 \| 0.30 \| 0.45 \| \| 17 to 24.9 \| 7.5 \| 0.30 \| 0.44 \| \| 25 to 35.9 \| 7.5 \| 0.21 \| 0.30 \| \| 36 to 59.9 \| 15 \| 0.25 \| 0.42 \| \| 60 to 79.9 \| 15 \| 0.19 \| 0.25 \| \| 80 to 90 \| 15 \| 0.17 \| 0.19 \| |

# ASPYR – full line.

|  |
| --- |
| \| **Weight** \| **Dose** \| **mg/kg** \| \| \| --- \| --- \| --- \| --- \| \| 5 to 7.9 \| 2.5 \| 0.32 \| 0.50 \| \| 8 to 14.9 \| 3.75 \| 0.25 \| 0.47 \| \| 15 to 19.9 \| 5 \| 0.25 \| 0.33 \| \| 20 to 23.9 \| 7.5 \| 0.31 \| 0.38 \| \| 24 to 44.9 \| 7.5 \| 0.17 \| 0.31 \| \| 45 to 64.9 \| 15 \| 0.23 \| 0.33 \| \| 65 to 90 \| 15 \| 0.17 \| 0.23 \| |

# ASPYR – 5 mg replaces 3.5 mg.

|  |
| --- |
| \| **Weight** \| **Dose** \| **mg/kg** \| \| \| --- \| --- \| --- \| --- \| \| 5 to 7.9 \| 2.5 \| 0.32 \| 0.50 \| \| 8 to 14.9 \| 5 \| 0.36 \| 0.63 \| \| 15 to 19.9 \| 5 \| 0.25 \| 0.33 \| \| 20 to 23.9 \| 7.5 \| 0.31 \| 0.38 \| \| 24 to 44.9 \| 7.5 \| 0.17 \| 0.31 \| \| 45 to 64.9 \| 15 \| 0.23 \| 0.33 \| \| 65 to 90 \| 15 \| 0.17 \| 0.23 \| |

# ASPYR – 7.5 mg replaces 5 mg in band 3.

|  |
| --- |
| \| **Weight** \| **Dose** \| **mg/kg** \| \| \| --- \| --- \| --- \| --- \| \| 5 to 7.9 \| 2.5 \| 0.32 \| 0.50 \| \| 8 to 14.9 \| 3.75 \| 0.25 \| 0.47 \| \| 15 to 19.9 \| 7.5 \| 0.38 \| 0.50 \| \| 20 to 23.9 \| 7.5 \| 0.31 \| 0.38 \| \| 24 to 44.9 \| 7.5 \| 0.17 \| 0.31 \| \| 45 to 64.9 \| 15 \| 0.23 \| 0.33 \| \| 65 to 90 \| 15 \| 0.17 \| 0.23 \| |

#

# Triple ALAQ – using 3.75 mg.

|  |
| --- |
| \| **Weight** \| **Dose** \| **mg/kg** \| \| \| --- \| --- \| --- \| --- \| \| 5 to 9.9 \| 2.5 \| 0.25 \| 0.50 \| \| 10 to 15.9 \| 3.75 \| 0.24 \| 0.38 \| \| 16 to 29.9 \| 7.5 \| 0.25 \| 0.47 \| \| 30 to 54.9 \| 15 \| 0.27 \| 0.50 \| \| 55 to 90 \| 15 \| 0.17 \| 0.27 \| |

# Triple ALAQ – 5 mg replaces 3.75 mg.

|  |
| --- |
| \| **Weight** \| **Dose** \| **mg/kg** \| \| \| --- \| --- \| --- \| --- \| \| 5 to 9.9 \| 2.5 \| 0.25 \| 0.50 \| \| 10 to 15.9 \| 5 \| 0.31 \| 0.50 \| \| 16 to 29.9 \| 7.5 \| 0.25 \| 0.47 \| \| 30 to 54.9 \| 15 \| 0.27 \| 0.50 \| \| 55 to 90 \| 15 \| 0.17 \| 0.27 \| |

# ASAQ – using 3.75 mg.

|  |
| --- |
| \| **Weight** \| **Dose** \| **mg/kg** \| \| \| --- \| --- \| --- \| --- \| \| 4.5 to 8.9 \| 2.5 \| 0.28 \| 0.56 \| \| 9 to 17.9 \| 3.75 \| 0.21 \| 0.42 \| \| 18 to 35.9 \| 7.5 \| 0.21 \| 0.42 \| \| 36 to 90 \| 15 \| 0.17 \| 0.42 \| |

# ASAQ – 5 mg replaces 3.75 mg.

|  |
| --- |
| \| **Weight** \| **Dose** \| **mg/kg** \| \| \| --- \| --- \| --- \| --- \| \| 4.5 to 8.9 \| 2.5 \| 0.28 \| 0.56 \| \| 9 to 17.9 \| 5 \| 0.28 \| 0.56 \| \| 18 to 35.9 \| 7.5 \| 0.21 \| 0.42 \| \| 36 to 90 \| 15 \| 0.17 \| 0.42 \| |

# ASMQ – using 3.75 mg.

|  |
| --- |
| \| **Weight** \| **Dose** \| **mg/kg** \| \| \| --- \| --- \| --- \| --- \| \| 5 to 8.9 \| 2.5 \| 0.28 \| 0.50 \| \| 9 to 17.9 \| 3.75 \| 0.21 \| 0.42 \| \| 18 to 29.9 \| 7.5 \| 0.25 \| 0.42 \| \| 30 to 90 \| 15 \| 0.17 \| 0.50 \| |

# ASMQ – 5 mg replaces 3.75 mg.

|  |
| --- |
| \| **Weight** \| **Dose** \| **mg/kg** \| \| \| --- \| --- \| --- \| --- \| \| 5 to 8.9 \| 2.5 \| 0.28 \| 0.50 \| \| 9 to 17.9 \| 5 \| 0.28 \| 0.56 \| \| 18 to 29.9 \| 7.5 \| 0.25 \| 0.42 \| \| 30 to 90 \| 15 \| 0.17 \| 0.50 \| |

# AL – 5 mg in the second dosing band.

|  |
| --- |
| \| **Weight** \| **Dose** \| **mg/kg** \| \| \| --- \| --- \| --- \| --- \| \| 5 to 14.9 \| 2.5 \| 0.17 \| 0.5 \| \| 15 to 24.9 \| 5 \| 0.2 \| 0.33 \| \| 25 to 34.9 \| 7.5 \| 0.21 \| 0.3 \| \| 35 to 90 \| 15 \| 0.17 \| 0.43 \| |

# AL – 7.5 mg in the second dosing band.

|  |
| --- |
| \| **Weight** \| **Dose** \| **mg/kg** \| \| \| --- \| --- \| --- \| --- \| \| 5 to 14.9 \| 2.5 \| 0.17 \| 0.50 \| \| 15 to 24.9 \| 7.5 \| 0.30 \| 0.50 \| \| 25 to 34.9 \| 7.5 \| 0.21 \| 0.30 \| \| 35 to 90 \| 15 \| 0.17 \| 0.43 \| |

#

# Vivax-matched regimen – using 3.75 mg.

This regimen uses 15 mg for the highest weight band.

|  |
| --- |
| \| **Weight** \| **Dose** \| **mg/kg** \| \| \| --- \| --- \| --- \| --- \| \| 5 to 7.9 \| 2.5 \| 0.32 \| 0.50 \| \| 8 to 16.9 \| 3.75 \| 0.22 \| 0.47 \| \| 17 to 40.9 \| 7.5 \| 0.18 \| 0.44 \| \| 41 to 80.9 \| 15 \| 0.19 \| 0.37 \| \| 81 to 90 \| 15 \| 0.17 \| 0.19 \| |

#

# Vivax-matched regimen – 5 mg replaces 3.75 mg.

This regimen uses 15 mg for the highest weight band.

|  |
| --- |
| \| **Weight** \| **Dose** \| **mg/kg** \| \| \| --- \| --- \| --- \| --- \| \| 5 to 7.9 \| 2.5 \| 0.32 \| 0.50 \| \| 8 to 16.9 \| 5 \| 0.30 \| 0.63 \| \| 17 to 40.9 \| 7.5 \| 0.18 \| 0.44 \| \| 41 to 80.9 \| 15 \| 0.19 \| 0.37 \| \| 81 to 90 \| 15 \| 0.17 \| 0.19 \| |

# Table S1. Modelled weight for age table.

| **Weight (kg)** | **Age (years)** | | | | |
| --- | --- | --- | --- | --- | --- |
|  | **min** | **p5** | **p50** | **p95** | **max** |
| 5 | 0.5 | 0.5 | 0.5 | 1 | 1 |
| 6 | 0.5 | 0.5 | 0.5 | 1 | 2 |
| 7 | 0.5 | 0.5 | 0.5 | 1 | 3 |
| 8 | 0.5 | 0.5 | 1 | 2 | 4 |
| 9 | 0.5 | 0.5 | 1 | 3 | 5 |
| 10 | 0.5 | 1 | 2 | 3 | 5 |
| 11 | 0.5 | 1 | 2 | 4 | 6 |
| 12 | 0.5 | 1 | 3 | 4 | 7 |
| 13 | 1 | 2 | 3 | 4 | 7 |
| 14 | 1 | 2 | 3 | 5 | 8 |
| 15 | 1 | 2 | 4 | 6 | 8 |
| 16 | 2 | 3 | 4 | 6 | 9 |
| 17 | 2 | 3 | 4 | 7 | 9 |
| 18 | 2 | 3 | 4 | 8 | 10 |
| 19 | 3 | 3 | 6 | 9 | 10 |
| 20 | 3 | 4 | 7 | 9 | 11 |
| 21 | 3 | 4 | 8 | 10 | 11 |
| 22 | 4 | 5 | 8 | 11 | 11 |
| 23 | 4 | 6 | 9 | 11 | 12 |
| 24 | 5 | 7 | 9 | 12 | 12 |
| 25 | 5 | 7 | 10 | 12 | 13 |
| 26 | 5 | 7 | 10 | 12 | 18 |
| 27 | 6 | 8 | 10 | 12 | 14 |
| 28 | 6 | 8 | 11 | 13 | 18 |
| 29 | 6 | 8 | 11 | 14 | 18 |
| 30 | 7 | 9 | 11 | 15 | 18 |
| 31 | 7 | 9 | 12 | 15 | 18 |
| 32 | 7 | 9 | 12 | 16 | 18 |
| 33 | 8 | 10 | 12 | 18 | 18 |
| 34 | 8 | 10 | 13 | 18 | 18 |
| 35 | 9 | 10 | 15 | 18 | 18 |
| 36 | 8 | 11 | 15 | 18 | 18 |
| 37 | 9 | 12 | 16 | 18 | 18 |
| 38 | 10 | 12 | 17 | 18 | 18 |
| 39 | 10 | 13 | 18 | 18 | 18 |
| 40 | 9 | 14 | 18 | 18 | 18 |
| 41 | 10 | 15 | 18 | 18 | 18 |
| 42 | 10 | 15 | 18 | 18 | 18 |
| 43 | 10 | 15 | 18 | 18 | 18 |
| 44 | 11 | 15 | 18 | 18 | 18 |
| 45 | 11 | 15 | 18 | 18 | 18 |
| 46 | 11 | 15 | 18 | 18 | 18 |
| 47 | 12 | 15 | 18 | 18 | 18 |
| 48 | 12 | 15 | 18 | 18 | 18 |
| 49 | 12 | 15 | 18 | 18 | 18 |
| 50 | 12 | 15 | 18 | 18 | 18 |
| 51 | 13 | 15 | 18 | 18 | 18 |
| 52 | 13 | 16 | 18 | 18 | 18 |
| 53 | 13 | 16 | 18 | 18 | 18 |
| 54 | 13 | 16 | 18 | 18 | 18 |
| 55 | 14 | 16 | 18 | 18 | 18 |
| 56 | 14 | 16 | 18 | 18 | 18 |
| 57 | 14 | 16 | 18 | 18 | 18 |
| 58 | 14 | 16 | 18 | 18 | 18 |
| 59 | 15 | 16 | 18 | 18 | 18 |
| 60 | 15 | 16 | 18 | 18 | 18 |
| 61 | 15 | 17 | 18 | 18 | 18 |
| 62 | 16 | 18 | 18 | 18 | 18 |
| 63 | 16 | 18 | 18 | 18 | 18 |
| 64 | 16 | 18 | 18 | 18 | 18 |
| 65 | 17 | 18 | 18 | 18 | 18 |
| 66 | 17 | 18 | 18 | 18 | 18 |
| 67 | 17 | 18 | 18 | 18 | 18 |
| 68 | 17 | 18 | 18 | 18 | 18 |
| 69 | 18 | 18 | 18 | 18 | 18 |
| 70 | 18 | 18 | 18 | 18 | 18 |
| 80 to < 90 | 18 | 18 | 18 | 18 | 18 |
| 90 to < 100 | 18 | 18 | 18 | 18 | 18 |

# Table S2. Age weight table.

| **Age (years)** | **Weight (kg)** | | | | |
| --- | --- | --- | --- | --- | --- |
|  | **min** | **p5** | **p50** | **p95** | **max** |
| 0.5 | 4.23 | 5.7 | 7.6 | 9.8 | 12.4 |
| 1 | 5.49 | 7 | 9.2 | 11.9 | 15.6 |
| 2 | 6.84 | 8.6 | 11.2 | 14.3 | 18.2 |
| 3 | 7.8 | 10 | 13 | 16.5 | 21 |
| 4 | 8.76 | 11.4 | 14.7 | 18.6 | 23.9 |
| 5 | 9.98 | 12 | 15.5 | 19.7 | 26 |
| 6 | 11.4 | 13.7 | 17.4 | 22 | 29.6 |
| 7 | 12.9 | 15.1 | 19.5 | 25 | 32.1 |
| 8 | 14.7 | 17 | 21.3 | 27.6 | 36 |
| 9 | 16.7 | 18.9 | 23.5 | 30.4 | 40.6 |
| 10 | 18.7 | 20.8 | 25.8 | 34 | 43 |
| 11 | 20.8 | 23 | 28.2 | 37 | 46 |
| 12 | 23 | 24.8 | 30.7 | 40.2 | 50.6 |
| 13 | 25.3 | 27.5 | 34.3 | 46.4 | 54 |
| 14 | 27.3 | 30 | 37 | 51 | 58 |
| 15 | 29.4 | 35.2 | 47 | 57.9 | 61 |
| 16 | 31.4 | 38.4 | 49.5 | 61 | 64.8 |
| 17 | 33.3 | 40.4 | 51.6 | 64 | 68.4 |
| 18 | 26.6 | 43.3 | 56.3 | 78.9 | 121.8 |
